# Supplementary material for: Global, regional, and cryptic population structure in a high gene-flow transatlantic fish
Source: PLoS One. 2023 Mar 20;18(3):e0283351. doi: 10.1371/journal.pone.0283351 (PMC10027230; doi:10.1371/journal.pone.0283351)

**PCA and DAPC plots**, all divisions as used in hierarchical clustering.

**The whole data, PCA (139 SNPs). 1597 fish from 39 locations.**


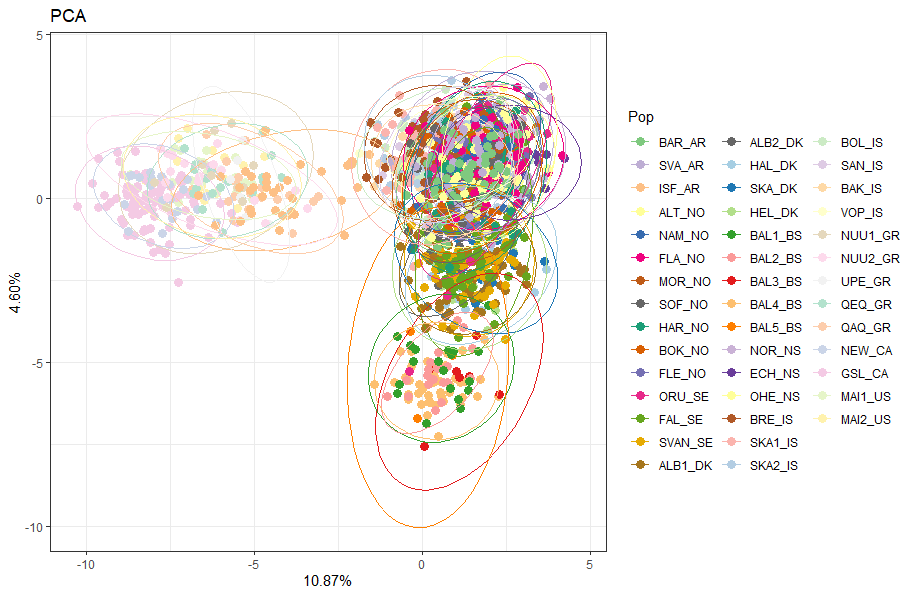


DAPC (130 PCs retained, 9 discriminant functions)


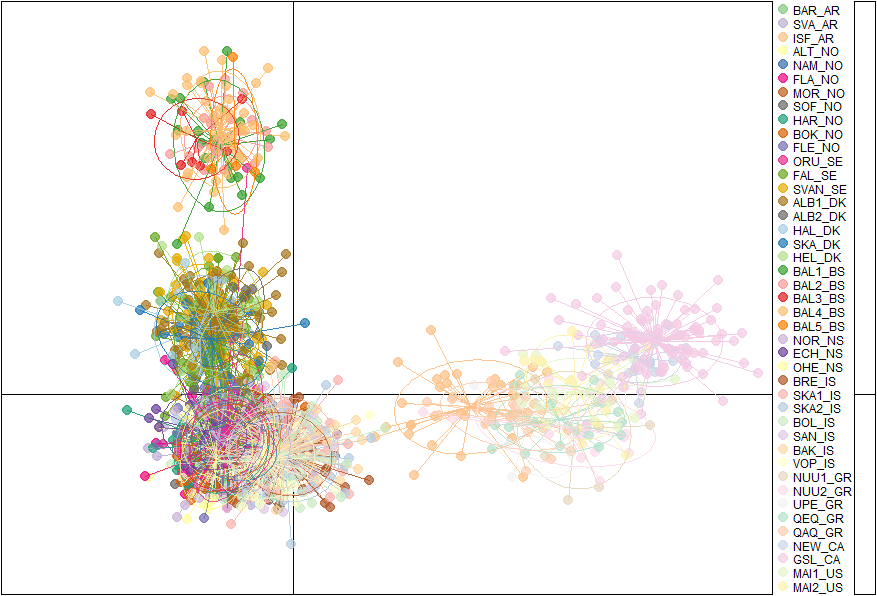


**The whole data, PCA (4393 SNPs). 95 fish from 10 locations.**


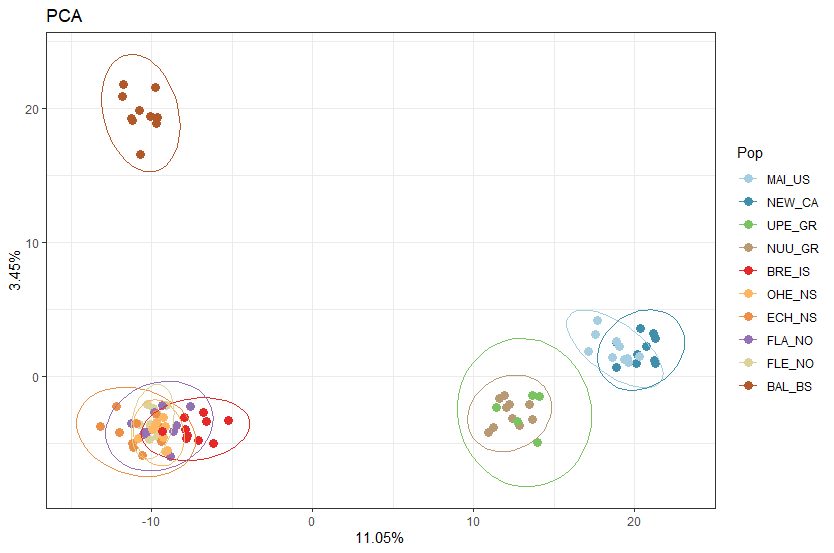


DAPC (90 PCs and 9 discriminant functions)


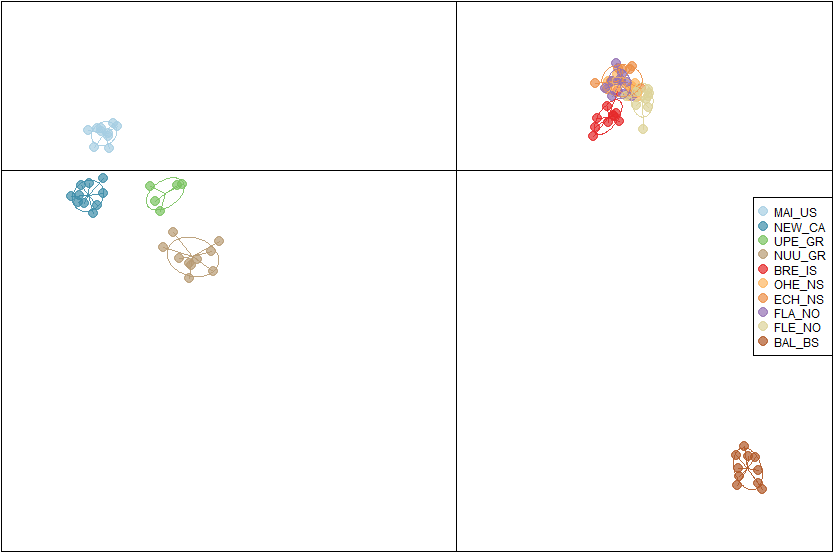


**The targeted data, PCA (139 SNPs). Same samples as the whole-genome dataset. 95 fish from 10 locations.**


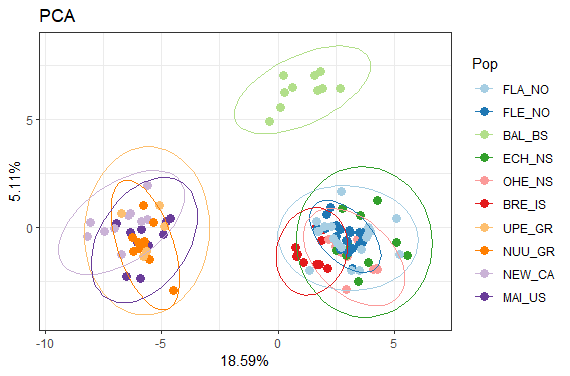


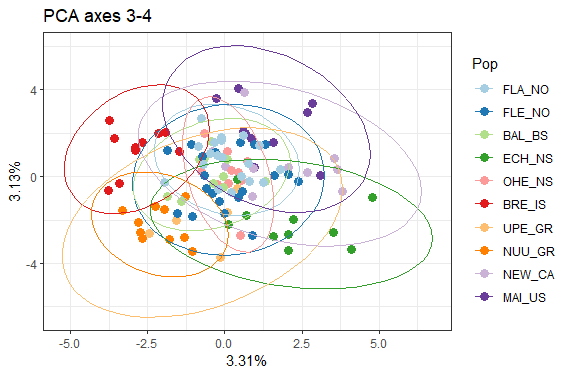


**Greenland and North America. (139 SNPs). 218 fish from 7 locations.**


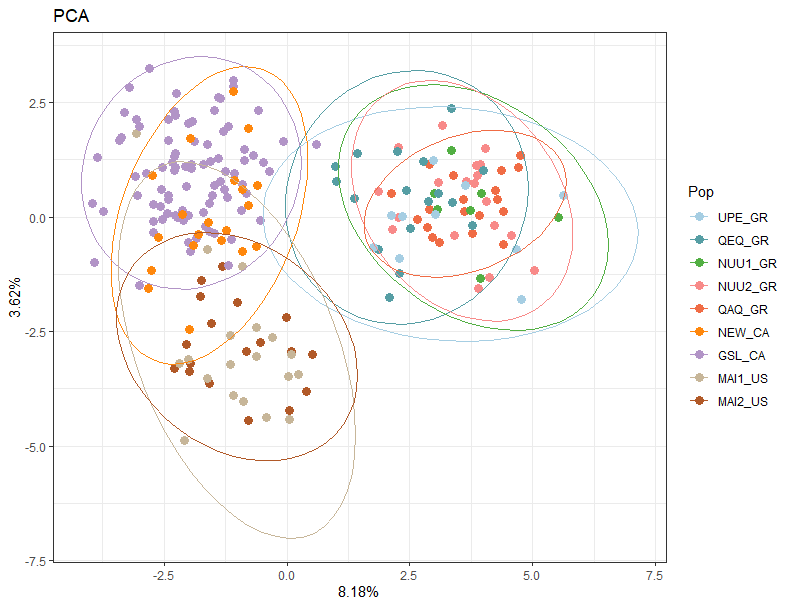


DAPC (60 PCs, 8 discriminant functions)


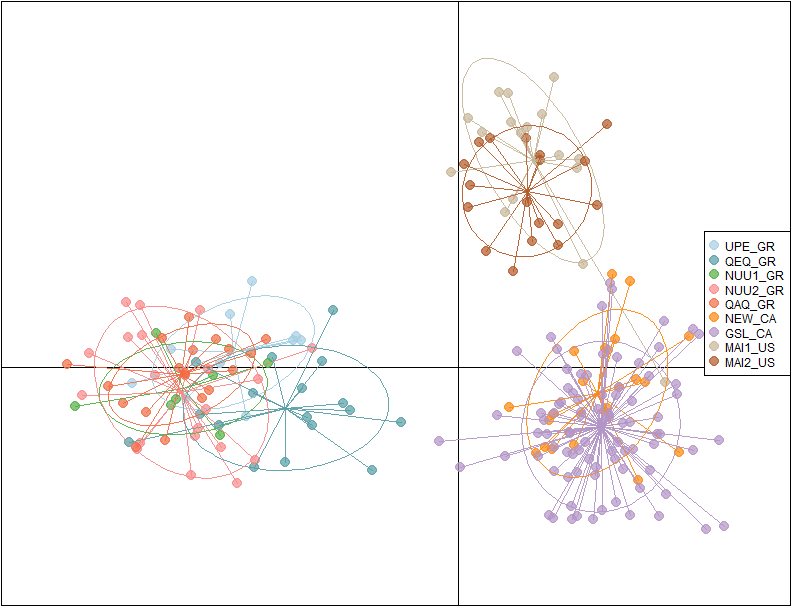


**PCA Iceland (139 SNPs). 386 fish from 6 locations.**


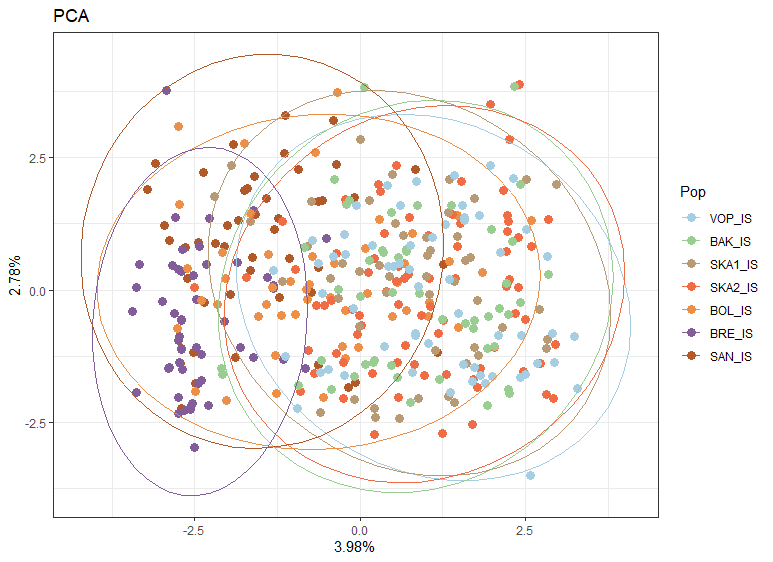


DAPC Iceland (80PCs, 6 discriminant functions)


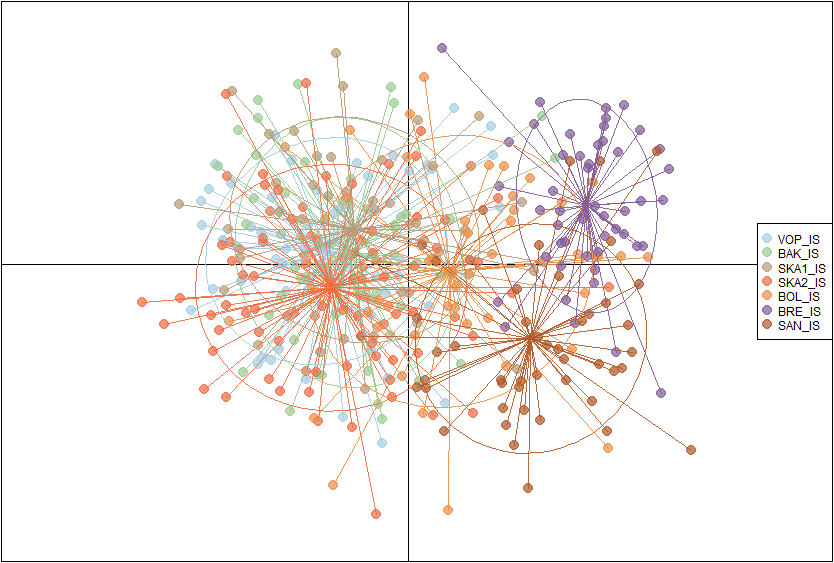


**Norway. (139 SNPs). 354 fish from 8 locations.**


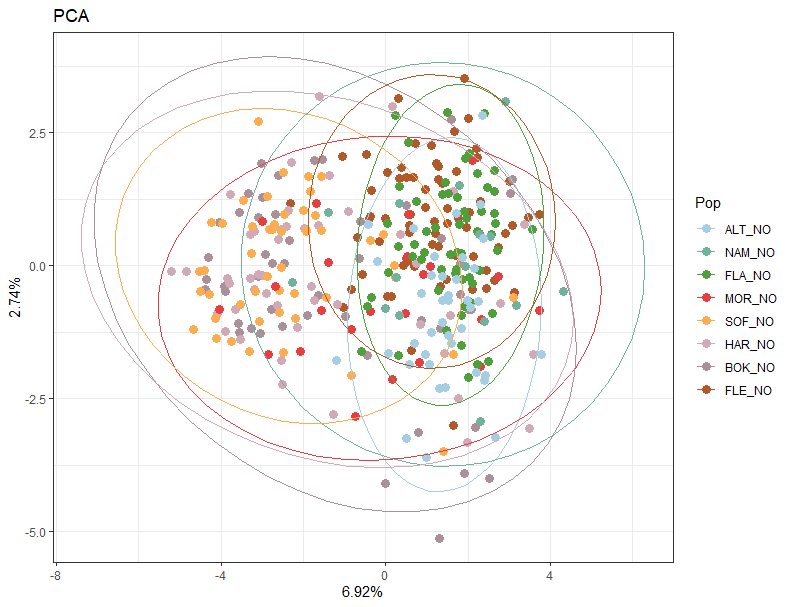


DAPC (80 PCs, 7 discriminant functions)


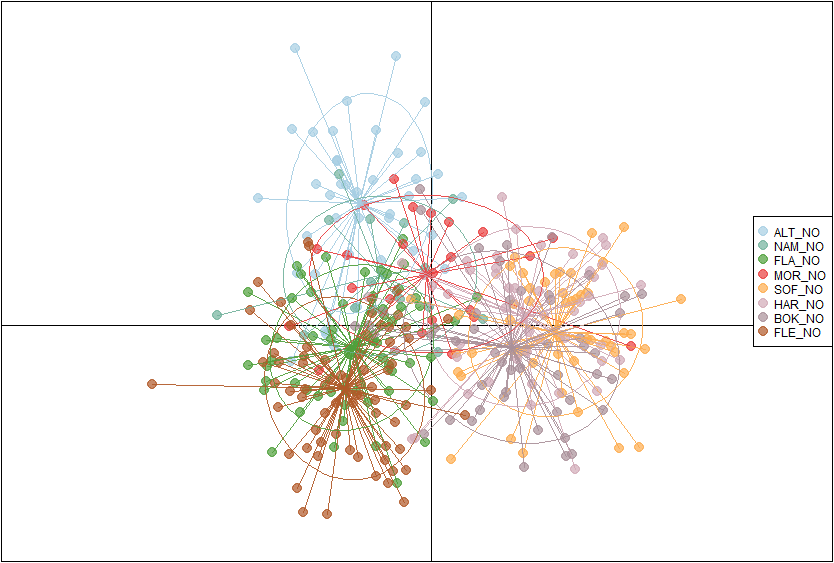


**Skagerrak, Kattegat, Baltic Sea. (139 SNPs). 451 fish from 12 locations.**


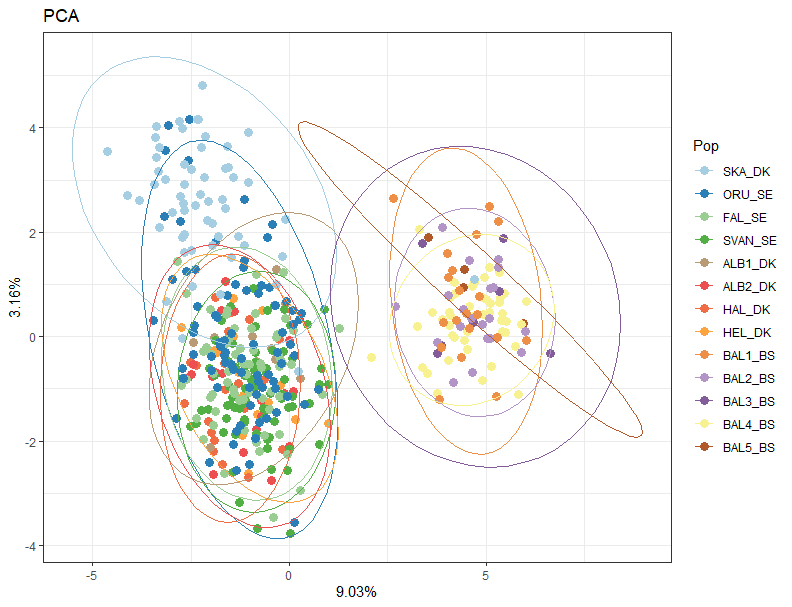


DAPC (120 PCs, 12 discriminant functions)


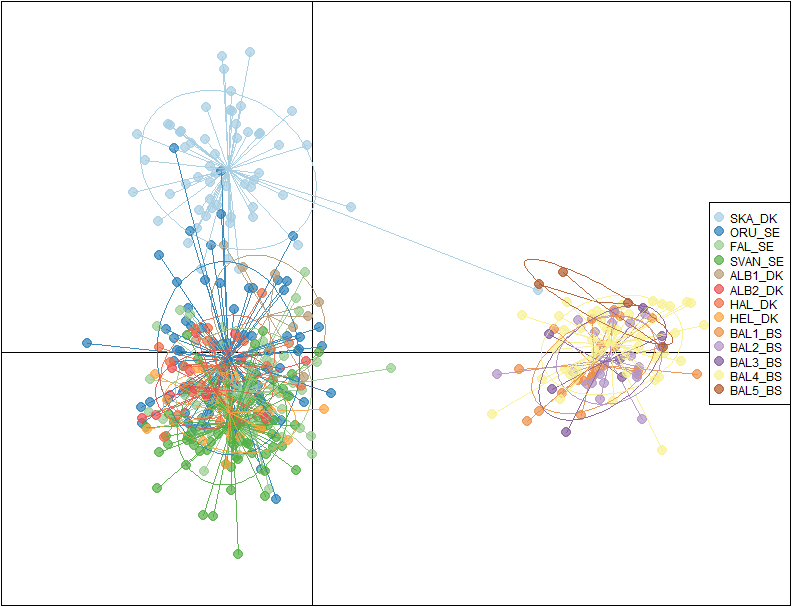


**Similar European populations with no clear assignment into any single population. (139 SNPs). 373 fish from 9 locations.**


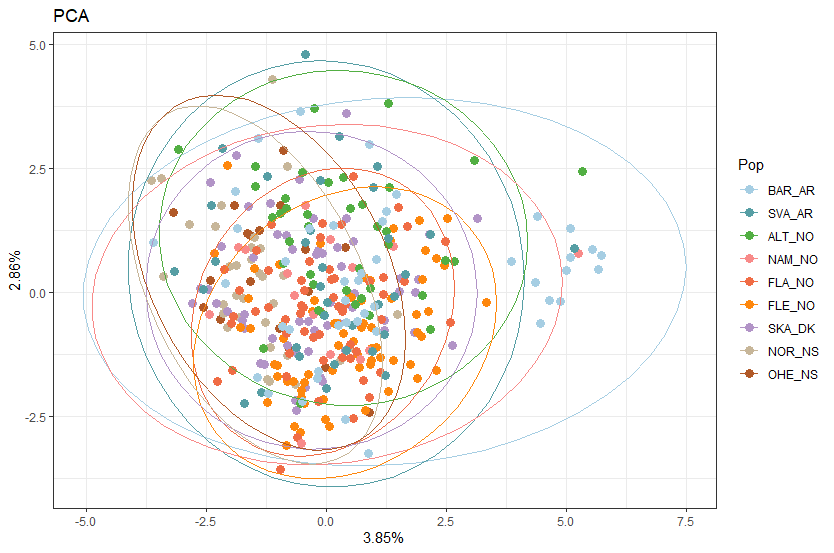


DAPC (50PCs, 8 discriminant functions)


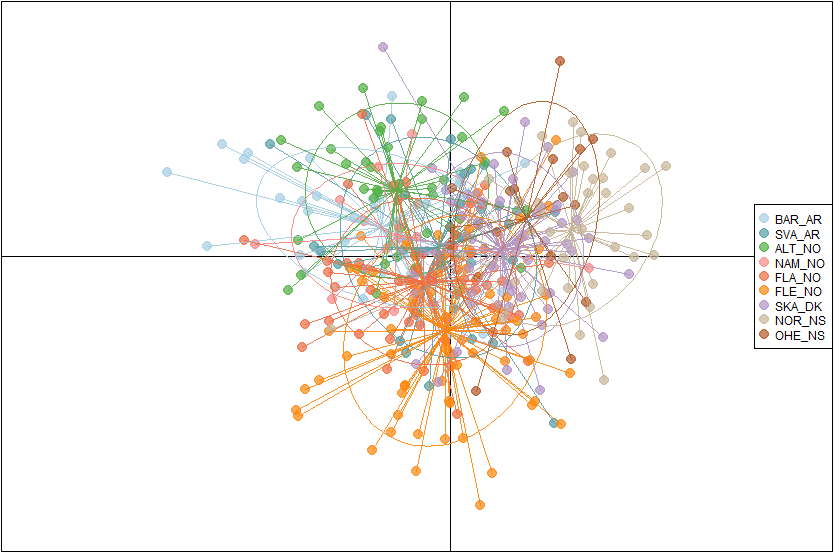


**Arctic sampling sites without Isfjorden (139 SNPs). 193 fish from 5 locations**

**
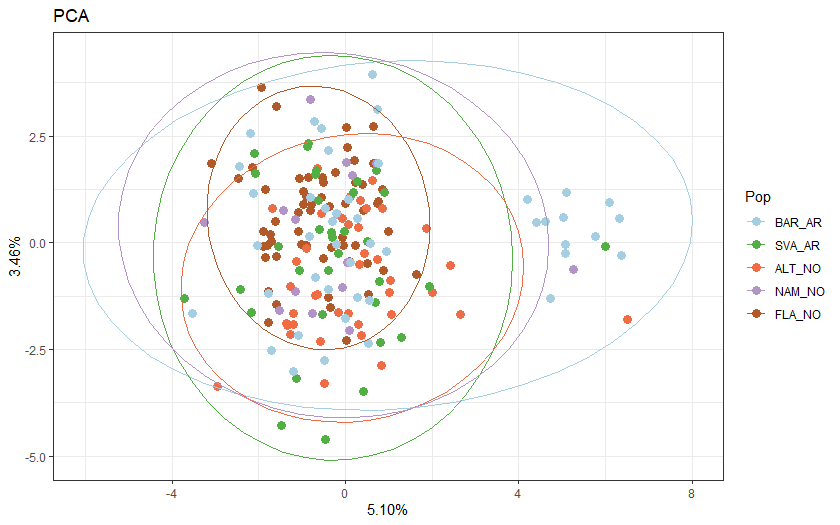
**

DAPC (40 PCs, 4 discriminant functions)


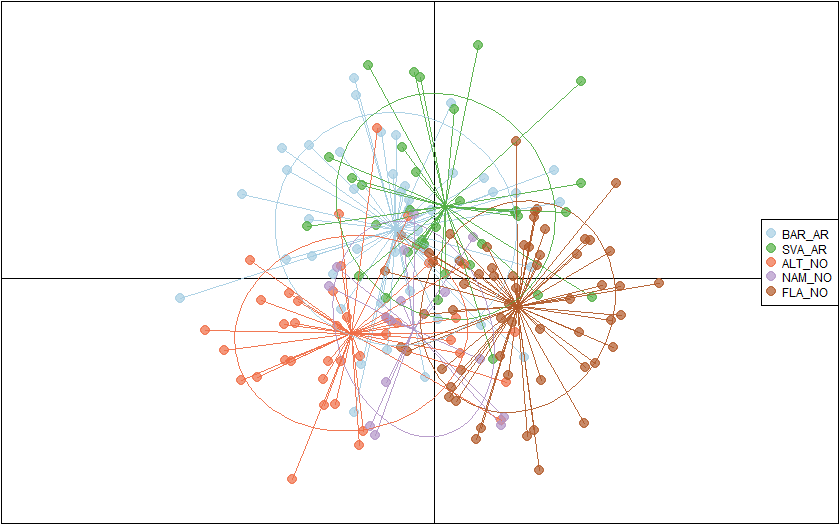

Supplement: S5 File — (DOCX) [file pone.0283351.s006.docx]
