# Supplementary material for: Global, regional, and cryptic population structure in a high gene-flow transatlantic fish
Source: PLoS One. 2023 Mar 20;18(3):e0283351. doi: 10.1371/journal.pone.0283351 (PMC10027230; doi:10.1371/journal.pone.0283351)

**LFMM2 Outliers**

SNPs which were detected as outliers connected to environmental variables using *LFMM* method and the reduced dataset of 139 SNPs. Below all analysis steps with results are shown.

**Step 1: Collinearity check.** When significant correlation (-0.7≤ *R* ≥ 0.7) between a pair of variables was detected, the variables in question were fused into the same synthetic variable.
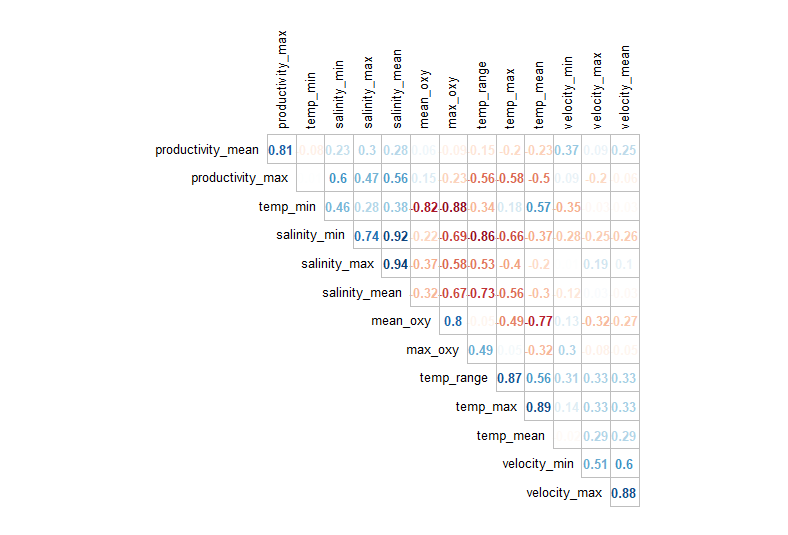


**Step 2: Regrouped correlated variables, the stability of created synthetic (combined) variables (5) and the correlation between them.**

**
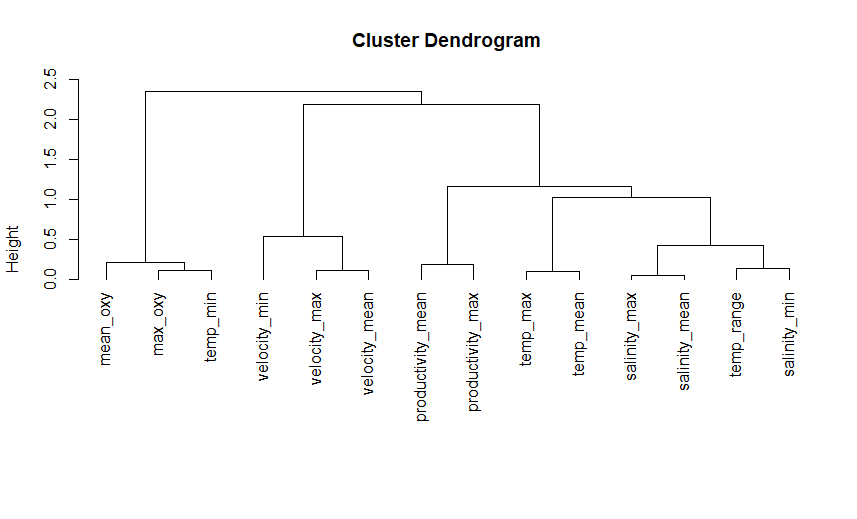
**

**
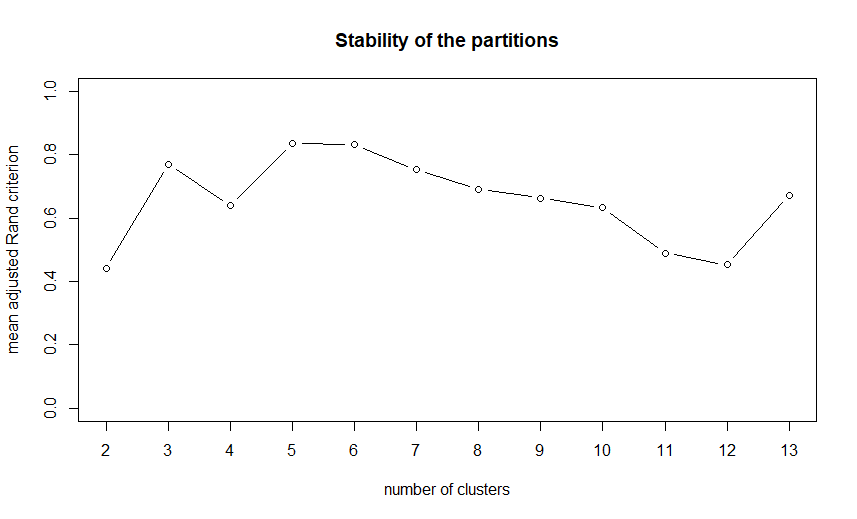
**

**
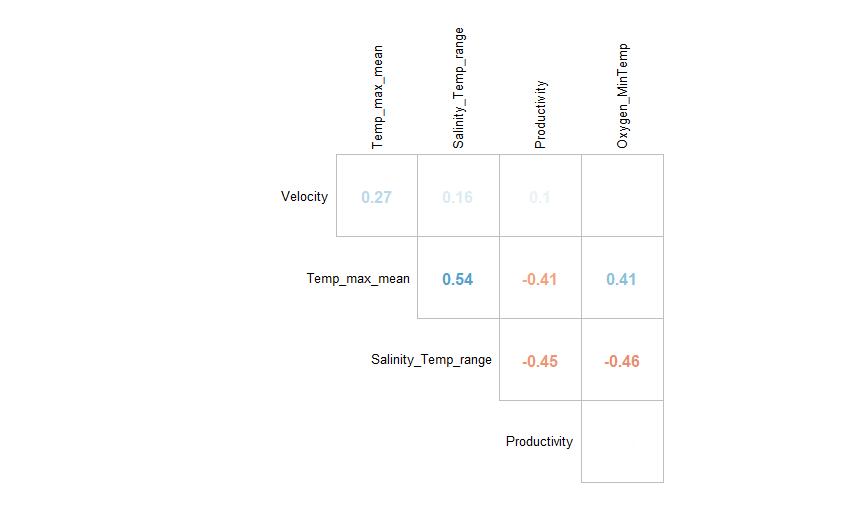
**

**Step 3. LFMM analysis between these synthetic variables and imputed genetic dataset**. Based on preliminary PCA, *K* was set to 6. Distribution of p-values for all SNP-environment associations.


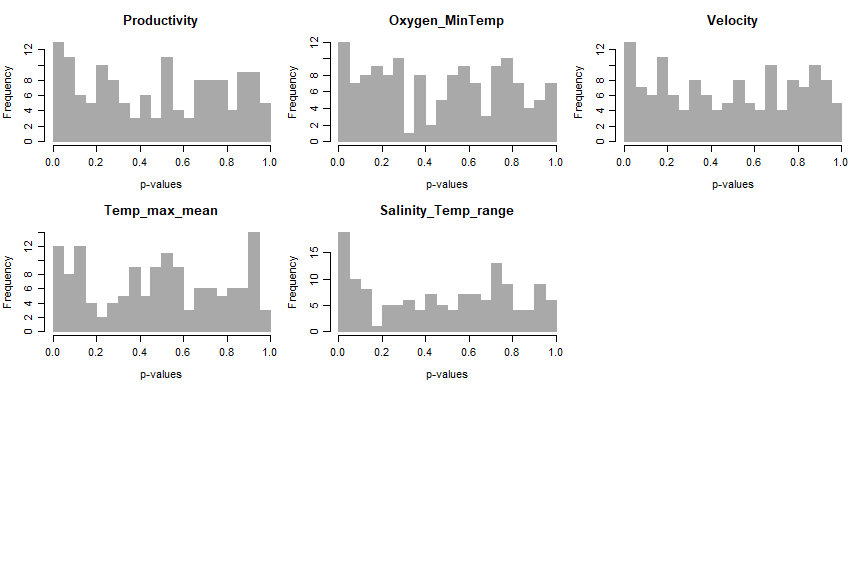


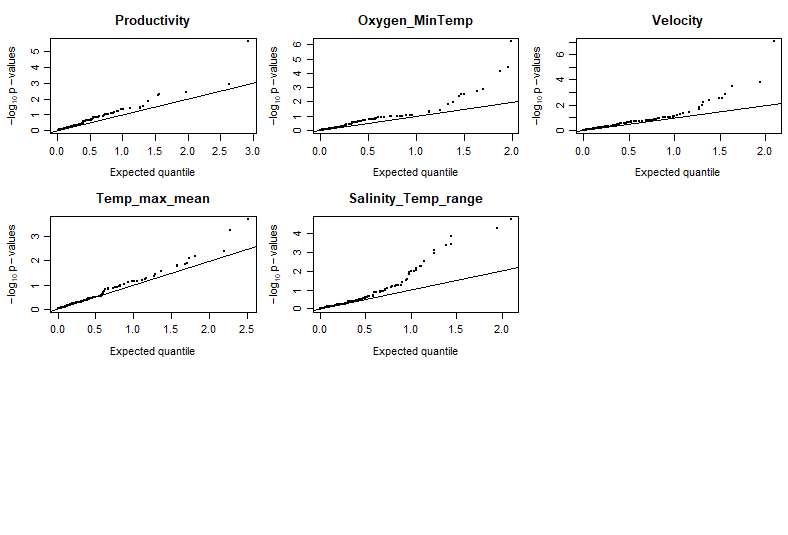


**Step 4. Derived q values from p values with false discovery rate (FDR).** Cut off of 5% was used as a threshold for significance. Below are q value distribution for each synthetic variable and a table of significant SNPs using a FDR threshold of 5%. A list of SNPs below the selected threshold is given in a table below.


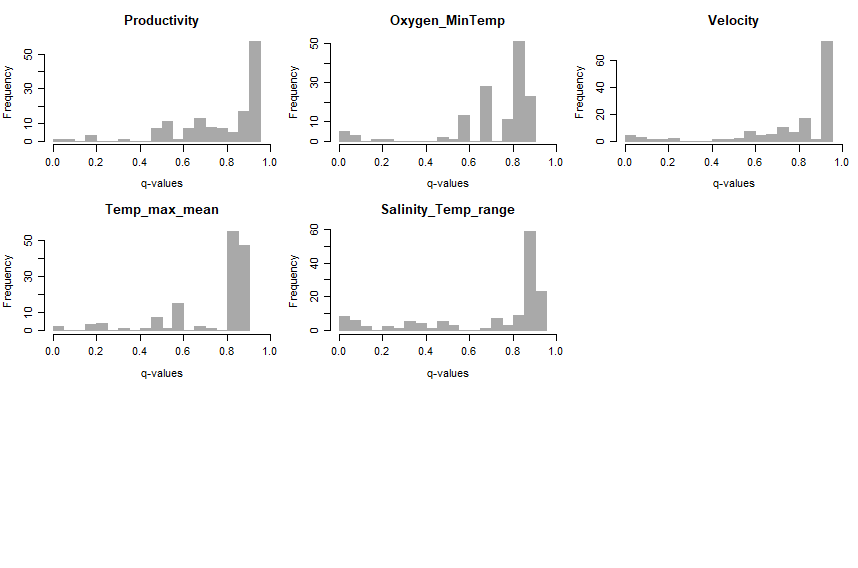

**Step 5. Visualization of allelic distribution ofthe putatively selected loci divided into larger regions.** Populations are in the same order as in Table 1 but VES_NO is removed, temporal replicates are combined and all Baltic Sea samples are combined into one. Note that only one allele from each locus is shown.


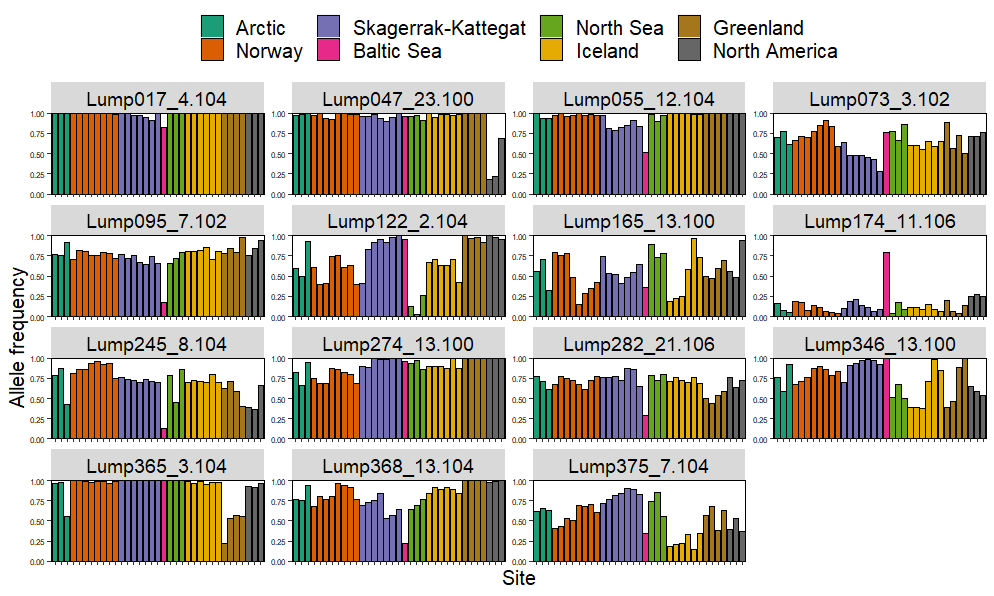

Supplement: S6 File — (DOCX) [file pone.0283351.s007.docx]
