# Supplementary material for: Global, regional, and cryptic population structure in a high gene-flow transatlantic fish
Source: PLoS One. 2023 Mar 20;18(3):e0283351. doi: 10.1371/journal.pone.0283351 (PMC10027230; doi:10.1371/journal.pone.0283351)
Supplement: Attachment — Submitted filename: PONE-D-22-29672_reviewer1.pdf [file pone.0283351.s016.pdf]

PLOS ONE

Global, regional, and cryptic population structure in a high gene-flow transatlantic fish

--Manuscript Draft--

**Manuscript Number:**	PONE-D-22-29672
**Article Type:**	Research Article
**Full Title:**	Global, regional, and cryptic population structure in a high gene-flow transatlantic fish
**Short Title:**	Population structure in Lumpfish
**Corresponding Author:**	Ellika Faust, Ph.D<br>University of Gothenburg Faculty of Science: Goteborgs universitet Naturvetenskapliga Fakulteten<br>Strömstad, SWEDEN
**Keywords:**	lumpsucker; Cyclopterus lumpus; SNP; outlier detection; genotype-environment association; 2b-RAD sequencing; cleaner fish; population genomics; seascape genetics; SNP panel; population genetics; fisheries; aquaculture; coastal; marine ecology
**Abstract:**	Lumpfish (Cyclopterus lumpus) is a transatlantic marine fish displaying large population sizes and a high potential for dispersal and gene-flow. These features are expected to result in a weak population structure. Here, we investigated population genetic structure of lumpfish throughout its natural distribution in the North Atlantic using two approaches: I) 4,393 genome wide SNPs and 95 individuals from 10 locations, and II) 139 discriminatory SNPs and 1,669 individuals from 40 locations. Both approaches identified extensive population genetic structuring with a major split between the East and West Atlantic and a distinct Baltic Sea population, as well as further differentiation of lumpfish from the English Channel, Iceland, and Greenland. The targeted discriminatory loci displayed ~2-5 times higher divergence than the genome wide approach, revealing further evidence of local population substructures. Lumpfish from Isfjorden in Svalbard were highly distinct but resembled most fish from Greenland. The Kattegat area in the Baltic transition zone, formed a previously undescribed distinct genetic group. Also, further subdivision was detected within North America, Iceland, West Greenland, Barents Sea, and Norway. Although lumpfish have considerable potential for dispersal and gene-flow, the observed high levels of population structuring throughout the Atlantic suggests that this species displays natal homing and potentially local populations with adaptive differences. This fine-scale population structure calls for consideration when defining management units for lumpfish fishing stocks and in decisions related to sourcing and moving lumpfish for farming and cleaner fish use.
**Order of Authors:**	Eeva Jansson
	Ellika Faust, Ph.D
	Dorte Bekkevold
	María Quintela
	Caroline Durif
	Kim Tallaksen Halvorsen
	Geir Dahle
	Christophe Pampoulie
	James Kennedy
	Benjamin Whittaker
	Laila Unneland
	Søren Post
	Carl André

Powered by Editorial Manager® and ProduXion Manager® from Aries Systems Corporation

	Kevin Glover
**Additional Information:**	
**Question**	**Response**
**Financial Disclosure**<br><br>Enter a financial disclosure statement that describes the sources of funding for the work included in this submission. Review the submission guidelines for detailed requirements. View published research articles from *PLOS ONE* for specific examples.<br><br>This statement is required for submission and **will appear in the published article** if the submission is accepted. Please make sure it is accurate.<br><br>**Unfunded studies**<br>Enter: *The author(s) received no specific funding for this work.*<br><br>**Funded studies**<br>Enter a statement with the following details:<br>• Initials of the authors who received each award<br>• Grant numbers awarded to each author<br>• The full name of each funder<br>• URL of each funder website<br>• Did the sponsors or funders play any role in the study design, data collection and analysis, decision to publish, or preparation of the manuscript?<br>• **NO** - Include this sentence at the end of your statement: *The funders had no role in study design, data collection and analysis, decision to publish, or preparation of the manuscript.*<br>• **YES** - Specify the role(s) played.<br><br>* typeset	This study was funded by the Norwegian Ministry for Trade, Industry and Fisheries, the Swedish research council FORMAS and the European Regional Development Fund (Interreg project “Margen II”)<br>The funders had no role in study design, data collection and analysis, decision to publish, or preparation of the manuscript.
**Competing Interests**<br><br>Use the instructions below to enter a competing interest statement for this submission. On behalf of all authors, disclose any competing interests that could be perceived to bias this work—acknowledging all financial support and any other relevant financial or non-financial competing interests.	The authors have declared that no competing interests exist.

Powered by Editorial Manager® and ProduXion Manager® from Aries Systems Corporation

This statement is required for submission and **will appear in the published article** if the submission is accepted. Please make sure it is accurate and that any funding sources listed in your Funding Information later in the submission form are also declared in your Financial Disclosure statement.<br><br>View published research articles from *PLOS ONE* for specific examples.<br><br>**NO authors have competing interests**<br><br>Enter: *The authors have declared that no competing interests exist.*<br><br>**Authors with competing interests**<br><br>Enter competing interest details beginning with this statement:<br><br>*I have read the journal's policy and the authors of this manuscript have the following competing interests: [insert competing interests here]*<br><br>* typeset	
**Ethics Statement**<br><br>Enter an ethics statement for this submission. This statement is required if the study involved:<br><br>• Human participants<br>• Human specimens or tissue<br>• Vertebrate animals or cephalopods<br>• Vertebrate embryos or tissues<br>• Field research<br><br>Write "N/A" if the submission does not require an ethics statement.<br><br>General guidance is provided below. Consult the submission guidelines for detailed instructions. **Make sure that all**	Ethical review and approval was not required for the animal study because This is a common commercial<br>species and the fish used in this study were not killed specifically to enable this study. All samples in this study were either taken<br>from fish which were already sampled for other studies or taken from already dead fish caught commercially or for other<br>surveys. In each country involved, national laws and regulations were strictly followed.

information entered here is included in the Methods section of the manuscript.<br><br>**Format for specific study types**<br><br>**Human Subject Research (involving human participants and/or tissue)**<br>• Give the name of the institutional review board or ethics committee that approved the study<br>• Include the approval number and/or a statement indicating approval of this research<br>• Indicate the form of consent obtained (written/oral) or the reason that consent was not obtained (e.g. the data were analyzed anonymously)<br><br>**Animal Research (involving vertebrate animals, embryos or tissues)**<br>• Provide the name of the Institutional Animal Care and Use Committee (IACUC) or other relevant ethics board that reviewed the study protocol, and indicate whether they approved this research or granted a formal waiver of ethical approval<br>• Include an approval number if one was obtained<br>• If the study involved *non-human primates*, add *additional details* about animal welfare and steps taken to ameliorate suffering<br>• If anesthesia, euthanasia, or any kind of animal sacrifice is part of the study, include briefly which substances and/or methods were applied<br><br>**Field Research**<br><br>Include the following details if this study involves the collection of plant, animal, or other materials from a natural setting:<br>• Field permit number<br>• Name of the institution or relevant body that granted permission	
**Data Availability**<br><br>Authors are required to make all data underlying the findings described fully available, without restriction, and from the time of publication. PLOS allows rare exceptions to address legal and ethical	Yes - all data are fully available without restriction

Powered by Editorial Manager® and ProduXion Manager® from Aries Systems Corporation

concerns. See the PLOS Data Policy and FAQ for detailed information.<br><br>A Data Availability Statement describing where the data can be found is required at submission. Your answers to this question constitute the Data Availability Statement and **will be published in the article**, if accepted.<br><br>**Important:** Stating 'data available on request from the author' is not sufficient. If your data are only available upon request, select 'No' for the first question and explain your exceptional situation in the text box.<br><br>Do the authors confirm that all data underlying the findings described in their manuscript are fully available without restriction?	
**Describe where the data may be found in full sentences. If you are copying our sample text, replace any instances of XXX with the appropriate details.**<br><br>• If the data are **held or will be held in a public repository**, include URLs, accession numbers or DOIs. If this information will only be available after acceptance, indicate this by ticking the box below. For example: *All XXX files are available from the XXX database (accession number(s) XXX, XXX.*).<br>• If the data are all contained **within the manuscript and/or Supporting Information files**, enter the following: *All relevant data are within the manuscript and its Supporting Information files.*<br>• If neither of these applies but you are able to provide **details of access elsewhere**, with or without limitations, please do so. For example:<br><br>*Data cannot be shared publicly because of [XXX]. Data are available from the XXX Institutional Data Access / Ethics Committee (contact via XXX) for researchers who meet the criteria for access to confidential data.*	All raw sequence files are available from the SRA database (accession number PRJNA858951)<br>Genotype files and metadata vill be uploaded to dryad data upon acceptance.

Powered by Editorial Manager® and ProduXion Manager® from Aries Systems Corporation

The data underlying the results presented in the study are available from (include the name of the third party and contact information or URL).<br>• This text is appropriate if the data are owned by a third party and authors do not have permission to share the data.<br><br>* typeset	
Additional data availability information:	Tick here if the URLs/accession numbers/DOIs will be available only after acceptance of the manuscript for publication so that we can ensure their inclusion before publication.

Manuscript
Click here to access/download;Manuscript;Lumpfish_PlosOne_19.10.2022.d

Global, regional, and cryptic population structure in a high gene-flow transatlantic fish

Eeva Jansson[1#], Ellika Faust[2#*], Dorte Bekkevold[3], María Quintela[1], Caroline Durif[4], Kim Tallaksen Halvorsen[5], Geir Dahle[1], Christophe Pampoulie[6], James Kennedy[6], Benjamin Whittaker[7], Laila Unneland[1], Søren Post[8], Carl André[2¤], Kevin A. Glover[1,9¤]

[#] These authors share first authorship
¤ These authors share last authorship
* Corresponding author: email: ellika.faust@gu.se

[1]Institute of Marine Research, P. O. Box 1870 Nordnes, N-5817 Bergen, Norway
[2]Department of Marine Sciences - Tjärnö Marine Laboratory, University of Gothenburg, 45296 Strömstad, Sweden
[3]DTU-Aqua National Institute of Aquatic Resources, Technical University of Denmark, Silkeborg, Denmark
[4]Institute of Marine Research, Austevoll Research Station, N-5392, Storebø, Norway
[5]Institute of Marine Research, Flødevigen, Nye Flødevigveien 20, N-4817 His, Norway
[6]Marine and Freshwater Research Institute, Fornubúðir 5, 220 Hafnarfjörður, Iceland
[7] Department of Biosciences, Centre for Sustainable Aquatic Research, Swansea University, Swansea, United Kingdom
[8]Greenland Institute of Natural Resources, Kivioq 2, Nuuk, Greenland
[9]Institute of Biology, University of Bergen, P. O. Box 7803, N-5020 Bergen, Norway

Keywords: *lumpsucker, Cyclopterus lumpus, SNP, outlier, genotype-environment association, 2b-RAD sequencing, cleaner fish, population genomic, seascape genetics*

1 Abstract

Lumpfish (*Cyclopterus lumpus*) is a transatlantic marine fish displaying large population sizes and a high potential for dispersal and gene-flow. These features are expected to result in a weak population structure. Here, we investigated population genetic structure of lumpfish throughout its natural distribution in the North Atlantic using two approaches: I) 4,393 genome wide SNPs and 95 individuals from 10 locations, and II) 139 discriminatory SNPs and 1,669 individuals from 40 locations. Both approaches identified extensive population genetic structuring with a major split between the East and West Atlantic and a distinct Baltic Sea population, as well as further differentiation of lumpfish from the English Channel, Iceland, and Greenland. The targeted discriminatory loci displayed ~2-5 times higher divergence than the genome wide approach, revealing further evidence of local population substructures. Lumpfish from Isfjorden in Svalbard were highly distinct but resembled most fish from Greenland. The Kattegat area in the Baltic transition zone, formed a previously undescribed distinct genetic group. Also, further subdivision was detected within North America, Iceland, West Greenland, Barents Sea, and Norway. Although lumpfish have considerable potential for dispersal and gene-flow, the observed high levels of population structuring throughout the Atlantic suggests that this species displays natal homing and potentially local populations with adaptive differences. This fine-scale population structure calls for consideration when defining management units for lumpfish fishing stocks and in decisions related to sourcing and moving lumpfish for farming and cleaner fish use.

2 Introduction

Understanding population demographics and population genetic structure is important for effective management and sustainable exploitation of wild species. Globally, it has been estimated that 34% of the assessed fish stocks are currently overfished, and another 60% are fully utilized [1]. Stock assessments have traditionally been based on changes in abundance over time, but to be accurate, they need to account also for population genetic structure and connectivity patterns among populations, and their underlying drivers [2-4]. Therefore, and where available, knowledge of population genetic structure should be included in management regimes (5-7). This applies also to marine species that usually display wide distribution ranges and large census population sizes, factors that often are considered to lead to weak population structure [8]. Genetic studies on marine fish have shown that, I) the effective population size reflecting population resilience may be only a fraction of the census size [9,10], II) the rate of effective migration might be lower than anticipated also in species with pelagic eggs and larvae [11], III) populations often encompass local adaptations [12-15], and that, IV) existing management units often misalign with population genetic structure and barriers to gene-flow [6,16, 17]. Moreover, as populations are temporally and spatially dynamic entities, it is important that the status of species and their populations is monitored and regularly updated, especially when emerging methods and approaches can provide new insights [18-22].

Lumpfish, *Cyclopterus lumpus*, is a cold-water marine species [23] distributed throughout the North Atlantic [24] (Figure 1) that has a long history of commercial exploitation. Fishing occurs almost exclusively when lumpfish migrate to coastal areas to spawn. At first the fishery was only small-scale and targeted males to be salted, smoked or dried for human consumption [24, 25]. Later on, and towards the end of the 1940s, commercial scale

exploitation of lumpfish females for their roe was initiated, and is now amounting in many thousand tons per year [25]. Recently, smaller scale fisheries have been established to capture mature individuals to serve as broodstock for juvenile lumpfish production that are in turn used as cleaner fish to remove parasitic sea lice in salmonid aqua/mariculture [26-28]. The use of farmed lumpfish has been expanding rapidly in recent years, and in Norway alone, over 40 million lumpfish are sold annually as cleaner fish to salmonid farms (Supplementary Figure 1). This use of lumpfish in aquaculture raises several concerns. First, while the industry is gradually moving towards closed production cycles, the majority of broodfish are still of wild origin [28], adding pressure on wild populations with often unknown status. Second, escapes of translocated lumpfish from net pens in salmonid farms represent a potential threat to local lumpfish populations, such as introduction of foreign genes and/or diseases [29]. Escape of Atlantic salmon (*Salmo salar*) is very common [30,31], and escape of other species of cleaner fish from commercial salmon farms has already been documented [32-34].

Figure 1. Distribution map of sampling localities of lumpfish (*Cyclopterus lumpus*) across the North Atlantic Ocean. Blue background indicates the approximate distribution area for lumpfish. Triangles indicate samples with sequencing data that were used to explore genome-wide population genetic patterns and to develop SNP markers. Circles indicate samples with SNP genotype data only. Colors indicate region as described in Table 1. Fish from three mid-sea samples, the Barents Sea, Svalbard, and the North Sea, were collected on research cruises covering a wide area. For these, the point shown on the map is the midpoint of all collection sites (for details, see Supplementary Figures 2a-d).

Adult lumpfish are solitary and inhabit the upper 50m of the water column [24 in offshore waters. Lumpfish are generally found in low densities and spread over a large geographical area [35]. When the adults are ready to spawn, they migrate to shallow coastal waters. During this migration, they will make extensive vertical movements through the water column, and show a greater association with the sea bed [36]. Males arrive at the coast earlier

than females in order to seek out and establish a territory/nesting site. When females arrive, they deposit their eggs in the male´s nests, where the male fertilizes the eggs and the females leave quickly [37]. Males guard the eggs that they fertilized until they hatch. Following hatching, the larvae attach to substrates, including seaweed and floating seaweed clumps [38]. Juveniles remain in shallow water areas for approximately 6-12 months before gradually making their way to the feeding grounds offshore. Low recapture rates in tag-recapture studies [39] and limited age classes of spawning lumpfish [40] have led to the suggestion that post-spawning mortality is high and that they have adopted a semelparous life strategy. However, facultative iteroparity has been shown to be possible and females which return to spawn the following year, spawn at roughly the same time [41] and in the same area [42] as they did previously, in support of natal homing behavior.

Earlier studies of population genetic structure of lumpfish revealed the existence of three major genetic groups: 1) western Atlantic, including samples from USA, Canada and Greenland, 2) eastern Atlantic, including samples from Iceland and Norway, and 3) the Baltic [43-45]. It was suggested that the major genetic break between western and eastern Atlantic is formed by the cold polar currents going south, keeping populations separated, and that the Baltic Sea population likely became isolated after the colonization of the brackish Baltic Sea around 8,000 years ago [43]. Garcia-Mayoral et al. [44] found latitudinal differentiation along the West coast of Greenland consistent with an isolation-by-distance (IBD) model, and a genetic break between populations in Northwest and Southwest Greenland. In the eastern Atlantic, lumpfish from the English Channel and Iceland have been found to be genetically distant from each other and all other eastern Atlantic samples [45]. There is contradictory evidence on whether there is structuring among Norwegian lumpfish [45, 46]. So far, only

smaller sets of microsatellite loci have been utilized in population genetic studies of lumpfish across the North Atlantic, and often concentrating on one part of the distribution range.

In this study, we aim to improve our understanding of connectivity and population genetic structure of lumpfish across the entire North Atlantic, with a focus on the eastern Atlantic and the Norwegian coast where lumpfish are increasingly being used as cleaner fish, increasing the risk of human mediated gene-flow. In order to achieve this, we first identified a set of genome-wide SNP markers using 2b-RAD sequencing [47]. Thereafter, we selected a set of putatively discriminatory SNPs (hereon referred to as the targeted SNPs) to resolve regional population structure, to be used in a geographically much more comprehensive study. We genotyped a high number of individuals (N=1,669), collected over the entire species' distribution (Figure 1), including previously unexplored northernmost areas in the north-eastern Atlantic. Finally, both datasets were analyzed jointly with several environmental variables to identify patterns and drivers of local adaptation.

3 Material and methods

3.1 Sampling

In total, 1,669 lumpfish collected in the period 2008-2020 from 40 locations were included in this study (Figure 1; Table 1). For lumpfish collected over a larger area, the average latitude and longitude were used (Figure 1; Supplementary Figure 2a-d). Four locations had temporal replicates, which were treated as independent samples to investigate temporal genetic variation. Waterway distances among all sampling sites were determined with the least path method implemented in package *marmap* version 1.0.5 [48] in the R environment, version 4.0.3 [49].

Table 1. Information of samples ordered from east to west and from north to south. The coordinates are approximate. Sample codes shown with grey backgrounds are temporal replicates from the same location. Bold codes denote samples with both SNP and genome wide data. Baltic Sea samples were not considered temporal replicates as they were collected from a wider area as part of different surveys. N displays number of individuals after filtering, followed by the number of individuals which did not pass filtering in brackets.

			**Coordinates**				
**Location**	**Code**	**Region**	**Lat**	**Lon**	**N**	**Collection time**	**Type***
Barents Sea	BAR_AR	Arctic	74.760	28.774	45 (1)	August 2020	migratory
Svalbard, open sea	SVA_AR	Arctic	75.219	9.754	34	September 2020	migratory, 26 adults, 8 juveniles
Isfjorden, Svalbard	ISF_AR	Arctic	78.394	16.593	36	October 2019	all juveniles
Alta, Norway	ALT_NO	Norway	70.475	21.811	39	July 2019	34 adults, 5 juveniles
Vesterålen, Norway	VES_NO	Norway	68.992	15.218	1 (6)	May 2020	all adults
Namsen, Norway	NAM_NO	Norway	64.482	11.291	15	June 2019	12 adults, 3 juveniles
Flatanger, Norway	FLA_NO	Norway	64.505	10.676	59 (1)	March 2019	likely spawning
Møre, Norway	MOR_NO	Norway	63.347	8.362	23	May 2019	15 adults, 8 juveniles
Sognefjorden, Norway	SOF_NO	Norway	61.266	4.845	49 (1)	May 2019	31 adults, 19 juveniles
Hardangerfjorden, Norway	HAR_NO	Norway	60.060	5.143	48 (4)	May 2019	38 adults, 14 juveniles
Boknafjorden, Norway	BOK_NO	Norway	59.217	5.867	50	April 2019	21 adults, 29 juveniles
Flekkefjorden, Norway	FLE_NO	Norway	58.188	6.609	70	March 2019	likely spawning
Skagerrak (ICES SQ 45F9)	SKA_DK	Skagerrak/Kattegat	58.250	9.500	57	July 2019	migratory
Orust, Sweden	ORU_SE	Skagerrak/Kattegat	58.045	11.182	81	2019	all juveniles
Falkenberg, Sweden	FAL_SE	Skagerrak/Kattegat	56.462	12.308	66 (3)	2019	all likely adults
Svanshall, Sweden	SVAN_SE	Skagerrak/Kattegat	56.160	12.381	80	May 2019	all likely adults
Ålbæk, North Kattegat	ALB1_DK	Skagerrak/Kattegat	57.590	10.420	10	March 2018	all adults, spawning
Ålbæk, North Kattegat	ALB2_DK	Skagerrak/Kattegat	57.590	10.420	20	February 2020	all adults, spawning
Hals, Kattegat	HAL_DK	Skagerrak/Kattegat	56.970	10.330	22	March 2019	all adults, spawning
Helsingør, Øresund	HEL_DK	Skagerrak/Kattegat	56.060	12.593	18 (1)	February 2020	all adults, spawning
Baltic Sea, Bornholm Basin	BAL1_BS	Baltic Sea	54.957	15.288	20	2011-2012	>17cm 17, 12-17cm 3
Baltic Sea, Gotland Basin	BAL2_BS	Baltic Sea	57.950	18.970	20	2017	>17cm 10, 12-17cm 8, <12cm 2
Baltic Sea, Bornholm Basin	BAL3_BS	Baltic Sea	55.700	15.200	7	March 2020	>17cm 4, 12-17cm 3
Baltic Sea, Gotland Basin	BAL4_BS	Baltic Sea	58.174	18.003	46	October 2020	>17cm 1, 12-17cm 27, <12cm 18
Baltic Sea, Bornholm Basin	BAL5_BS	Baltic Sea	55.607	15.772	4	June 2020	>17cm 4
North Sea, mid sea	NOR_NS	North Sea/British Isles	59.020	0.462	35	August 2019	migratory

English Channel,British Isles	ECH_NS	North Sea/British Isles	50.042	-2.522	20	2015	adults, 9 males, 11 females
Outer Hebrides, British Isles	OHE_NS	North Sea/British Isles	58.160	-6.315	18 (2)	2017	all adults
Vopnafjörður, Iceland	VOP_IS	Iceland	65.777	-14.702	60	April 2019	all spawning females
Bakkafjörður, Iceland	BAK_IS	Iceland	66.166	-14.991	50	April 2011	all spawning females, 3-5 years
Skagastrønd, Iceland	SKA1_IS	Iceland	65.781	-20.461	50	February 2012	spawning adults, all males
Skagastrønd, Iceland	SKA2_IS	Iceland	65.781	-20.461	79 (1)	April 2019	spawning adults, all females
Bolungarvik, Iceland	BOL_IS	Iceland	66.158	-23.225	49 (1)	May 2012	spawning, 48 females, 2 males
Breiðafjörður, Iceland	BRE_IS	Iceland	65.235	-23.291	49	June 2011	adults, 4 males, 42 females, 3 NA
Sandgerði, Iceland	SAN_IS	Iceland	64.037	-22.773	49	May 2012	spawning, 48 females, 1 male
Upernavik, Greenland	UPE_GR	Greenland	72.790	-56.151	10	June 2014	spawning
Qeqertarsuaq, Greenland	QEQ_GR	Greenland	69.221	-53.698	17 (8)	June 2014	spawning
Nuuk, Greenland	NUU1_GR	Greenland	64.172	-51.764	7 (7)	2011	all adults, 6 females, 8 males
Nuuk, Greenland	NUU2_GR	Greenland	64.172	-51.764	20	June 2014	spawning
Qaqortoq, Greenland (South)	QAQ_GR	Greenland	60.737	-45.965	19 (6)	April 2015	spawning
Newfoundland, Canada	NEW_CA	North America	49.943	-63.910	20	June 2008	all spawning females
Gulf of St. Lawrence, Canada	GSL_CA	North America	49.429	-59.153	88 (1)	2019	54 adults, 35 juveniles
Maine (Scantum Basin), USA	MAI1_US	North America	42.833	-70.580	19 (1)	2012	9 females and 6 males, 3 juveniles, 2 NA
Maine (Scantum Basin), USA	MAI2_US	North America	42.833	-70.580	18 (1)	2019	all adults, 13 females, 5 males, 1 NA

*Fish from outside the Baltic Sea ≤330g and/or ≤20 cm were considered as juveniles. When sex, spawning status and/or age are known, they are listed. In the Baltic Sea, fish are divided into three size classes.

3.2 DNA isolation, sequencing and data filtering

Genomic DNA was extracted from fin clips stored in absolute ethanol with the Qiagen DNeasy Blood & Tissue Kit following the manufacturer's instructions. Ninety-five fish from 10 locations across the species distribution area (Figure 1; Table 1) were selected for the modified restriction-site-associated DNA sequencing, 2b-RAD [50]. Library preparations were performed as detailed in Faust et al. [33] with the addition of degenerate tags for removing PCR duplicates. Sequencing was done at SciLifeLab in Sweden on an Illumina NovaSeq platform. The resulting raw sequences are available on NCBIs Sequence Read Archive [*will be added later*]. PCR duplicates, adapter sequences and low-quality reads were removed, and the remaining sequences were mapped to the *C. lumpus* draft genome [51] with *bowtie2* [52]. SNPs were called with UnifiedGenotyper GATK [53]. Variant score quality was recalibrated (VQSR) using site identity across technical replicates as a training set. Sites with more than 10% missing data and a fraction of heterozygotes above 0.5 (possible lumped paralogs) were removed, leaving a total of 7,301 SNPs. After adding a minor allele frequency (MAF) filter of > 0.1 in the total dataset, and a minor allele count of at least two, a final dataset of 4,393 SNPs, was retrieved. This genome-wide dataset was used to investigate general global population genetic patterns of lumpfish, as well as to select a subset of discriminatory SNPs.

3.3 Selection of SNP markers and genotyping

To find regionally discriminatory markers, non-linked (i.e not in linkage disequilibrium) SNPs with high divergence (F_{ST}) were filtered from multiple pairwise comparisons. SNPs with F_{ST} values above 0.4 were selected from the pairwise comparisons between the North-East Atlantic (Icelandic, UK and Norwegian samples) and 1) Greenland, 2) North America,

and 3) the Baltic Sea. For separation among the Norwegian and Scottish samples and among all the North-East Atlantic samples, 200 and 100 SNPs with the highest F_{ST} estimates were chosen, respectively (Supplementary Tables 2a-b). As many SNPs showed high divergence in multiple comparisons, this resulted in a total of 393 SNPs to be used for primer design. F_{ST} for each SNP was calculated with *diveRsity* [54]. Primer design, amplification and genotype calling were done using the Agena MassARRAY iPLEX Platform as described by Gabriel et al. [55]. The final selected set of markers consisted of 198 SNPs divided into seven assay groups (Supplementary Tables 2a-c).

All 1,669 lumpfish along with 19 technical replicates (i.e., replicate individuals) were genotyped with the selected 198 SNPs. Each 384-plate contained two to four negative controls. Technical replicates were used to estimate genotyping consistency. A total of 59 loci did not amplify or produce a clear clustering pattern and were discarded (Supplementary Table 2a). Individual and locus-wise call rates were checked with R package *dartR* [56], and loci with ≥15% missing data (N=4) and individuals with ≥ 20% missing data (N=44), were discarded. In Vesterålen, a single fish passed the filtering steps, and this sampling site was removed from further analysis. The final dataset consisted thus of 1,597 lumpfish from 39 different sites, genotyped with 139 informative SNPs. Technical replicates did not show discrepancies besides a likely drop-out detected for one individual in three loci. The final targeted SNP dataset had a total of 2.34% missing data. The selected SNPs were distributed across all 25 chromosomes in the lumpfish genome (Supplementary Table 2a), and many of them were located within (N=64) or in the immediate vicinity (less than 2kb away; N=16) of known/annotated gene sequences.

3.4 Population genetic analysis

The genome-wide, sequence-derived dataset consisting of 95 fish from 10 locations and 4,393 SNPs, is hereafter referred to as the "genome-wide dataset". The genotype data from the final 139 SNP panel is referred to as the "targeted dataset". All analyses were conducted with both datasets unless otherwise stated. Besides analyzing samples, several analyses were carried out using larger pooled groups based on geographic regions (as given in Table 1). When applicable, grouping was made differently based on results received from the upstream analyses.

3.4.1 Genetic variation and its division

Expected and observed heterozygosity, allelic richness, and inbreeding coefficient (F_{IS}) were estimated with the *diveRsity* package [54] in R. Confidence intervals at 95% were calculated for the F_{IS} indices using 1,000 bootstraps. Deviation from Hardy-Weinberg expectations (HWE) was estimated both locus- and sample-wise with Fisher's exact probability test and complete enumeration using Genepop v.1.1.7 [57]. For the targeted dataset, the distribution of variation between geographic regions, between sampling sites (and their temporal replicates) within regions, and within samples was investigated with AMOVA in the R package poppr v.2.9.1 [58]. Statistical significance for the variance components was obtained with 999 permutations using the ade4 package, v.1.7-16 [59]. Pairwise F_{ST}-values [60] were calculated with StAMPP [61] in R. Corresponding 95% confidence intervals and p values were calculated with 2,000 bootstraps, and corrected with False discovery rate (FDR) [62] for multiple comparisons. Pairwise F_{ST} were visualized with UPGMA dendrogram with *as.dist()* and *hclust()* functions in 'stats' package in R.

3.4.2 Individual-based clustering

Three clustering approaches were employed to investigate and visualize the genetic differentiation among individuals. First, clustering was done with Principal Component Analysis (PCA) with the R package *ade4* [59, 63]. PCA is a multivariate exploratory approach without assumptions on populations or their boundaries. Next, the variation that maximized among-group differences was identified using *adegenet*s*'* v.2.1.3 [64,65] discriminant analysis of principal components (DAPC) with the *xvalDapc* function to choose the optimal number of principal components. The third method, STRUCTURE v.2.3.4 [66,67], is a model-based Bayesian clustering method that uses a predefined number of *K* clusters to estimate the posterior probability of each individual's genotype to originate from each cluster. All STRUCTURE runs were performed using the default admixture model with correlated allele frequencies and with location information given a priori [68]. A total of 50 000 MCMC (Markov Chain Monte Carlo) repetitions were used in each run after 20 000 repeats were discarded as burn-in. *K* was set from 1 to 10 or 12 (depending if whole or partial dataset was used, see below), and the number of iterations was set to 5. To determine the optimal *K*, bar plots were inspected visually and runs analyzed with the StructureSelector software [69]. The software summarizes results as the optimal Ln Pr(X|K) given by the STRUCTURE software and the ad hoc summary statistic ΔK [70], which identifies the uppermost level of population hierarchy. Moreover, the StructureSelector calculates MedMed, MedMean, MaxMed and MaxMean as Puechmaille [71] described. Results from the runs for the different values of K were averaged with CLUMPAK [72] using the LargeKGreedy algorithm and 2,000 repeats.

As higher levels of structure can effectively mask lower levels of structure [71] a hierarchical approach was employed. The above-mentioned clustering analyses were therefore performed

first for the whole dataset, followed by a separate analysis for sampling sites that were geographically close and showed similar admixture profiles. For each of the investigated regions, the most informative loci behind the observed structure were determined with the help of PCA loadings. The threshold for being informative was set to 0.1.

3.4.3 Population graphs

Genetic structure for the targeted SNP data was also analyzed with Population Graphs, a graph-theoretic approach that uses conditional genetic distance [73] as the response variable, which is primarily created by both gene-flow and shared ancestry. This approach has been shown to capture underlying demographic processes more accurately than methods based on pairwise estimates of genetic structure or various genetic distance metrics [74]. The identification of genetic covariance structure among populations is independent of evolutionary assumptions aiming to minimize Hardy–Weinberg and linkage disequilibrium within populations [73]. Population Graphs were constructed using the R packages *popgraph* and *gstudio* [75,76] and topological analyses were performed using the *igraph* package [77].

For Population Graph, graph distance was estimated as the minimal topological distance connecting pairs of sampling sites (nodes) where the distances between nodes in the network, based on genetic covariance, was evaluated in relation to physical separation of nodes on the landscape under a model of isolation by graph distance, IBGD [73]. A relatively small graph distance between spatially distant sites indicates long-distance gene-flow (extended edges). Conversely, geographical or ecological barriers that impede gene-flow relative to other localities with similar distances generate relatively high graph distances (compressed edges). Correlations and detection of extended and compressed edges were determined by regressing

geographic and graph distances. Analyses were conducted using 35 geographically-explicit locations after pooling temporal samples, and pooling all Baltic Sea samples in one.

3.4.4 Environmental association analyses

Environmental factors deemed likely to be important to lumpfish populations were filtered from the Bio-ORACLE database (https://www.bio-oracle.org; [78,79]) for the sampling sites (Supplementary Table 1a; Supplementary Figure 3a-c). The decision of which factors to include was primarily based on expert opinion (C. Dürif, personal communication) but also comprised of additional factors commonly included in similar analyses of other marine fish species. Collinearity between variable pairs was investigated with the R package *corrr* v.0.4.3 [80] and *corrplot* version 0.84 [81]. Highly correlated variables were merged into new synthetic variables (Supplementary Table 1b) using a hierarchical clustering method in the R package *ClustOfVar* v.1.1 [82] and 100 bootstraps were used to estimate the obtained grouping stability.

To detect signals of selection and adaptation to the local environment, datasets were analyzed with two independent methods (Supplementary Table 1b). First, latent factor mixed model (LFMM), a univariate association method between genotypic and environmental variable matrices was run using functions implemented in the R package *LEA*, v.3.2.0 [83]. Here, the optimal number of principal components explaining the genetic variation (*K*) was determined with the function *pca()* and by choosing the “knee” point in the associated scree plot. To get the required complete genotypes, missing data was imputed using the *snmf()* function with the selected *K,* default settings and 10 repetitions. Best run of K clusters was determined as the one having the lowest cross entropy. Then, using the imputed dataset, the *lfmm2()* and *lfmm2.test()* functions were used to estimate latent factors based on the exact least-square

approach, and their adjusted *p* values. Here, the default value was used for the lambda parameter, and a linear model was selected for the following *p* value estimation. For the obtained *p* values between each environmental variable and SNP, corresponding *q* value was calculated using the R package *qvalue*, v.2.22.0 [84]. The threshold for false discovery rate was set to 5%.

Further, the R package, *pcadapt* v.4.1.0 [85], was used for the genome-wide data to perform a SNP outlier scan to cross-validate the obtained results from the LFMM analysis. *pcadapt* is a non-constrained ordination method that like LFMM uses a PCA approach to define the underlying population structure prior to the outlier scan. The test for outliers is based on the correlations between genetic variation and the first *K* principal components. Same *K* as for the LFMM approach was used here, and the *q* value threshold was set to 5%.

Finally, for the targeted dataset, redundancy analysis (RDA; see e.g. [86]) was adopted. RDA is a linear multivariate ordination method that can simultaneously analyze multiple environmental variables and genetic markers. Here, the geographic distance matrix between all sampling sites was first transformed into corresponding dbMEMs (distance-based Moran's Eigenvector Maps) using *adespatial*, v.0.3-14 [87]. Significant geographic and environmental variables, explaining allele frequency variation across samples, were identified using the *forward.sel()* function. Only significant variables were included in the actual RDA analysis with the R package *vegan*, v.2.5-7 [88]. RDA was performed using the full model for regression, followed by a partial model where geographic location was controlled for. Variance inflation factor was checked to be below the recommended 10 for all variables, and permutation tests were repeated 1000 times.

4 Results

4.1 Genetic variation and its division

Expected heterozygosity over the 4,393 SNPs varied between 0.124 and 0.151 across samples (Supplementary Table 3). Observed heterozygosity estimates were consistently slightly higher, as expected for outlier loci, between 0.132-0.179. Heterozygosity measured with the targeted 139 SNP panel was higher, between 0.193 and 0.280 (H_e). In 13 samples, observed heterozygosity deviated significantly from expected heterozygosity, and F_{IS} was positive in nine of them.

Most samples were significantly differentiated from each other (Table 2), and all studied levels of population hierarchies displayed significant divisions (Table 3). None of the temporal replicates were significantly differentiated (Supplementary Table 4). Global mean F_{ST} was 0.094 (SD ± 0.057) for the genome-wide dataset, and slightly higher, 0.115 (SD ± 0.007) for the targeted dataset. Three major groups were clearly identified: The highly-differentiated West and East Atlantic ($F_{ST\ (genome\text{-}wide)}$ 0.087-0.154), and the Baltic Sea ($F_{ST\ (genome\text{-}wide)}$ >0.08 and >0.16 against West and East Atlantic, respectively). Estimates of genetic divergence among these three groups were consistently higher, about two to five-fold, for the targeted dataset (Table 2, Supplementary Table 4b).

Table 2. Pairwise F_{ST} values among samples of lumpfish (*Cyclopterus lumpus*) with both genome-wide and targeted SNPs. Below diagonal shows estimated F_{ST}s. The first value is from the genome-wide data (4,393 SNPs) and in parentheses from the targeted data (139 SNPs). Above diagonal shows corresponding p values based on 1,000 bootstraps. If only one value is given, both comparisons coincided with that value. Pairwise F_{ST} table including remaining samples from the targeted SNP data is given in Supplementary Table 4a.

	Norway		Baltic Sea	British Isles		Iceland	Greenland		North America	
	Flatanger	Flekkefjord	Bornholm	English Channel	Outer Hebrides	Breiðafjörður	Upernavik	Nuuk	Newfoundland	Maine
Flatanger		0.010 (0)	0	0	0.027 (0)	0	0	0	0	0
Flekkefjord	0.004 (0.007)		0	0	0.007 (0)	0	0	0	0	0
Bornholm	0.083 (0.173)	0.082 (0.162)		0	0	0	0	0	0	0
English Channel	0.025 (0.063)	0.024 (0.048)	0.099 (0.220)		0	0	0	0	0	0
Outer Hebrides	0.003 (0.016)	0.004 (0.022)	0.087 (0.198)	0.021 (0.055)		0	0	0	0	0
Breiðafjörður	0.013 (0.036)	0.015 (0.046)	0.089 (0.176)	0.035 (0.111)	0.012 (0.054)		0	0	0	0
Upernavik	0.106 (0.251)	0.113 (0.251)	0.179 (0.313)	0.134 (0.309)	0.112 (0.272)	0.099 (0.223)		0	0	0
Nuuk	0.103 (0.216)	0.105 (0.214)	0.160 (0.278)	0.124 (0.270)	0.104 (0.237)	0.087 (0.171)	0.021 (0.037)		0	0
Newfoundland	0.150 (0.269)	0.154 (0.264)	0.204 (0.309)	0.170 (0.314)	0.152 (0.291)	0.141 (0.235)	0.060 (0.106)	0.065 (0.092)		0
Maine	0.140 (0.216)	0.144 (0.219)	0.196 (0.282)	0.161 (0.274)	0.141 (0.232)	0.132 (0.186)	0.076 (0.107)	0.078 (0.099)	0.026 (0.032)	

All p values remain significant ($p<0.05$) after correction for multiple testing.

Table 3. Distribution of genetic variance of lumpfish (*Cyclopterus lumpus*) for 139 targeted SNPs. Regions are as given in Table 1

Variance source	d.f.	Sum of squares	Variance components	% of variation	*p* value
Among regions	7	12 122	4.3	10.4	<0.001
Among samples within regions	35	3 659	0.9	2.2	<0.001
Among individuals within samples	1 554	58 735	1.5	3.6	<0.001
Within individuals	1 597	55 634	34.8	83.8	

Besides these three major branches, the targeted SNP dataset also showed separation between North America (USA and Canada) and West Greenland/Svalbard-Isfjorden. Also, Kattegat in the Baltic transition zone, the English Channel, Iceland and three fjords in southwestern Norway showed separation from their neighboring samples (Figure 2). In the West Atlantic, US and Canadian samples differed from Greenland ($F_{ST} \geq 0.07$) and from each other with a genome-wide mean F_{ST} of ~ 0.026. Within Greenland, samples north and south of 65°N were genetically different ($F_{ST} \geq 0.021$).

Figure 2. Dendrogram of population pairwise F_{ST} estimated in of lumpfish (*Cyclopterus lumpus*) based on a targeted dataset with 139 SNPs. Sample codes are explained in Table 1.

In the Arctic, Isfjorden stood out as highly differentiated from the samples collected offshore from Svalbard and the Barents Sea area, as well as from northern Norway and Iceland (Supplementary Table 4a). Instead, Isfjorden shared a close resemblance with western Greenland (Figure 2). Lumpfish from the Baltic Sea formed a highly distinct genetic group. Fish collected in the English Channel also formed a distinct group. Among the remaining East Atlantic samples, lumpfish collected from the Kattegat area, Iceland, and southwestern Norwegian fjords (Sognefjorden, Hardangerfjorden and Boknafjorden) each formed separate clusters. Lumpfish from the rest of Norway, Outer Hebrides, North Sea, Skagerrak, and open Arctic waters (Svalbard and Barents Sea) formed one group with relatively low genetic divergence among samples (Figure 2), referred to as the East Atlantic Group below.

4.2 Individual-based clustering

Plotting population structure with PCA revealed similar large-scale division as pairwise F_{ST} estimates (Supplementary File 1). The first axis divided the East and West Atlantic in both

genome-wide and targeted datasets, and explained 11.1% and 10.9% of the total variation, respectively. The second axis separated the Baltic Sea samples from the rest and explained 3.45% and 4.60%, respectively. Using the DAPC approach and regional partitioning of the data showed similar results as the PCA but with clearer clustering (Supplementary File 1). Lumpfish from the USA and Canada formed separate clusters, as did fish from Greenland, with a North-South separation. Icelandic lumpfish showed a West-East separation. In mainland Norway, three overlapping clusters were formed, roughly separating northernmost Alta, from large fjords in southwestern Norway (Sognafjorden, Hardangerfjorden and Boknafjorden), and from mid- and south-Norway (Flatanger and Flekkefjord). The Baltic Sea and the North Sea transition zone, Skagerrak and Kattegat, separated in two three distinct clusters, with the exception of one likely migrant from the Baltic in the Skagerrak offshore sample. Within the East Atlantic group, weak separation could be seen between northernmost areas (Barents Sea, Svalbard and Alta), mainland Norway (Flatanger and Flekkefjord), and Skagerrak-North Sea (including Outer Hebrides).

The results from STRUCTURE were consistent with those from the PCA/DAPC approaches but provided additional insights. In the genome-wide dataset, the division between western and eastern Atlantic was clear, but many individuals also shared signs of admixture (Figure 3). In the East Atlantic, all lumpfish shared at least ~ 50% of their genetic information. K=7 was deemed as the best fit for the data (Supplementary Table 5), as this was the highest value creating visually discrete clusters, corresponding to six genetic clusters: North America, Greenland, Iceland, English Channel, Baltic Sea, and finally Norway and the Outer Hebrides which clustered together.

Figure 3. Assignment of lumpfish (*Cyclopterus lumpus*) into genetic K clusters for the genome-wide dataset with 4,393 SNPs (above, K=7) and targeted dataset with 139 SNPs (below, K=9). Each bar is one individual and the different colors represent the proportional

assignment to the different K clusters. Samples which were genotyped with both methods are connected with black lines.

The targeted SNP dataset revealed nine major clusters (*K*=9), showing an additional cluster in Kattegat, the Norwegian southwestern fjords and an east-west divergence across Iceland. Hierarchical clustering analysis of West Atlantic samples showed a clear separation between US and Canada, and between northwest and southwest Greenland (Figure 4; Supplementary Table 5). In the samples from USA-Maine, a possible migrant from Canada was observed together with a possible first-generation hybrid (also clearly visible in DAPC plot; S. File 1).

The Baltic Sea and the North Sea transition zone, Kattegat and Skagerrak, were separated into three clusters (Figure 4; Supplementary Table 5). One individual caught in Skagerrak was clearly a migrant of Baltic Sea origin, as also seen in the PCA and DAPC (Supplementary File 1). The northernmost samples from Kattegat (Orust) displayed a high proportion of assignments to Skagerrak. Moreover, individuals from western and eastern Kattegat formed separate clusters. Among lumpfish the Norwegian mainland fjords, two distinct genetic clusters could be observed (Figure 4; Supplementary Table 5). As seen in both PCA and DAPC analysis, several individuals from the large southwestern Norwegian fjords (Sognefjorden, Hardangerfjorden and Boknafjorden) clustered together, separating them from all other Norwegian lumpfish. Additional levels of genetic structuring separated the northernmost Norwegian samples to some degree. When only samples from the Arctic (open sea and northernmost Norwegian fjords) were considered, *K*=2 was supported, and an additional Arctic cluster within the Barents Sea was revealed (Figure 4; Supplementary Table 5).

Figure 4. Regional STRUCTURE assignment of lumpfish (*Cyclopterus lumpus*) based on targeted 139 SNPs. Square represents separate structure runs for different regions where a set of samples, which due to their genetic and geographic closeness were analyzed together.

Each pie chart shows one sample's assignment to the selected number of clusters (*K*) averaged across individuals. Note that some of the samples are included in multiple assignments but that Isfjorden and the English Channel samples were not included in any regional structure analysis due to their uniqueness.

4.3 Patterns of population connectivity

The distribution of spatial genetic structure in the targeted dataset suggested restricted gene-flow under a model of isolation by graph distance, that is, distances between nodes in the network of genetic covariance in relation to geographic distance between samples (IBGD; Spearman's rho=0.733, p<0.2 e-16; Supplementary Figure 4). *Popgraph* depicted a complex web where the 35 nodes (samples) were connected by 119 edges (out of the 595 possible ones; Figure 5 ; Supplementary Table 6). Out of the 119 edges, 27 were proportional, 38 were extended (i.e., more gene-flow than what would be expected given geographic distance), and 54 were compressed (i.e., less gene-flow than what would be expected given geographic distance). The majority, 83% of the compressed edges, indicated less gene-flow occurred within regions. Conversely, 87% of the extended edges connected different regions, and 11% of the extended edges occurred between Norwegian nodes in a range of 700-1500 km of geographic distance.

Figure 5. Population Graph representing the distribution of genetic covariance in the global lumpfish (*Cyclopterus lumpus*) population based on 139 SNPs. Nodes represent samples, and their relative sizes the amount of within-population genetic variance. Edges connecting the nodes represent the pattern of genetic covariance among populations: Dashed lines denote extended edges (i.e., more gene-flow than what would be expected given geographic distance), dotted lines compressed edges, (i.e., less gene-flow than what would be expected given geographic distance), and continuous line proportional edges (i.e., not significantly more or less gene-flow).

4.4 Outlier locus detection and association with environment

Based on the correlation analysis among the 14 environmental factors, five independent synthetic variables (Productivity, Oxygen_MinTemp, Velocity, Temp_max_mean, and

Salinity_Temp_range; Supplementary Table 1b) were created for each sample and used further (see Supplementary File 2 for details). Using LFMM, we identified 58 significant SNP outlier locus – environmental variable associations in the genome-wide SNP data (Supplementary Table 7a-b). PCadapt detected a total of 24 outlier loci out of which nine were shared between the PCadapt and LFMM (Supplementary Table 7c). The identified outliers were distributed throughout the 25 lumpfish chromosomes. Most of the outlier loci were located within or in immediate vicinity of annotated gene sequences (Supplementary Table 7b-c). For the targeted dataset, LFMM identified 20 significant associations for 15 different SNPs (Supplementary Table 8; Supplementary File 2). Five of the SNPs were located within genes, two of which were also outliers in the genome-wide dataset.

Finally, based on redundancy analysis, four canonical axes and two synthetic environmental variables (Salinity/Temperature range, Oxygen level/Minimum Temperature) were significantly associated ($p < 0.001$) with the observed patterns of genetic structure. Together they explained 55.5% of the total variation (Figure 6). Partial analysis removing the effect of geography still showed a significant association ($R^2 = 0.086$, $p < 0.001$), albeit much weaker. Outliers were defined as being at least 2.5 standard deviations away from the mean SNP loadings along the significant axes. Only a single SNP (Lump-165) passed that criterion. The same SNP was denoted as a putative outlier in the LFMM analysis with both datasets (Supplementary Table 7b; Supplementary Table 8).

Figure 6. Seascape redundancy analysis of lumpfish (*Cyclopterus lumpus*). Moran Eigenvector's Maps (MEMs) decompose spatial relationships among samples based on a spatial weighting matrix. Four MEMs, and two synthetic environmental variables (Salinity/Temperature range, Oxygen level/Minimum Temperature) were significantly associated ($p < 0.001$) with the observed genetic structure. Together they explained 55.5% of the total variation. Samples are color-coded based on their geographic region, with all Baltic Sea samples pooled as one. Isfjorden and Skagerrak samples are labeled as separated from other samples in their region. Small blue circles denote 139 SNPs.

5 Discussion

The patterns of population genetic structure that we observe today results from a complex interplay of evolutionary forces from both the past and present. For lumpfish, strong oceanic currents, demographic history, homing behavior, glacial refugia, and local adaptation have all been suggested as likely drivers of genetic population structure [43-45]. Based on our study, lumpfish populations display extensive global, regional, as well as local patterns of population genetic structure, which are created by different processes and partly mediated by different parts of the genome. Combining genome-wide SNPs with a targeted SNP panel and extensive sampling, we uncovered multiple layers of population genetic structuring across the distribution of lumpfish. Complex patterns of connectivity among and within samples, and an overall genetic structure that was significantly linked with environmental variability, suggest that populations are locally adapted. For a widely distributed marine fish that undertakes offshore feeding migrations, natal homing is a likely mechanism for finer-scale genetic differentiation and evolution of local adaptation [89].

5.1 Global population structure

The two datasets, i.e., the 4,393 genome-wide SNPs and the targeted panel of 139 SNPs, revealed similar patterns for lumpfish concerning higher-level population structure: We observed clear genetic splits dividing West and East Atlantic, the Baltic Sea, Iceland and the English Channel. These large-scale patterns are concordant with the results from previous population genetic studies [43-45], and likely represent population patterns shaped by demographic history and strong oceanic currents. Similar deep genetic splits across the Atlantic Ocean have been described in other transatlantic species (e.g., Atlantic cod (*Gadus morhua)* [90] and red alga (*Palmaria palmata*) [91]). The split of these species likely

occurred >100 000 years ago during the interglacial period, when shallow ocean depths on continental shelves favored transcontinental movement and population expansions.

We found that the global population subdivision of lumpfish based on the genome-wide data alone would best be represented by six groups: North America, Western Greenland, Iceland, English Channel, Baltic Sea, and Norway, together with the Outer Hebrides. While using the targeted dataset and applying a hierarchical approach, we identified additional structuring with a total of nine major genetic groups globally: North America, Greenland together with Isfjorden in Svalbard, eastern and western Iceland split in two, English Channel, Baltic Sea, Kattegat in the North Sea transition zone, fjords in southwestern Norway and finally, an East Atlantic group covering a larger geographic area and including samples from Norway, Arctic open waters, Skagerrak, North Sea and the Outer Hebrides.

5.2 Regional population structures

We detected different degrees of sub-structuring within the large regional groups. Some finer-scale population patterns for lumpfish have already been described in previous studies where isolation-by-distance was detected in West Greenland [44], and also suggested in Norway where Averøy in mid-Norway was found to be different from other Norwegian samples [45]. Here we found further structuring within regions that have not been previously described. When we examined the genetic results jointly with geographic location and information on life history stages (Table 1), we found that samples with only breeding and juvenile lumpfish formed regional and clear-cut groups, whereas migrating fish, mid-sea samples and samples known to contain both juveniles and adults were more admixed. Genetic divergence of regional breeding populations is thus in line with observations which suggest natal homing in lumpfish [41, 42].

5.2.1 West Atlantic

In the western Atlantic, we found significant genetic differentiation among samples collected from Greenland, Canada and USA, as well as a north-south division along Greenland's west-coast, as described previously [43, 44]. Furthermore, the targeted SNP panel produced distinct clustering patterns for the groups (Figure 4; Supplementary Table 5), suggesting good regional resolution, and could likely be used to e.g., detect migrants. Indeed, two individuals collected in Maine, USA, had high admixture proportions (~50 and ~80%) and resembled the Canadian sample, suggesting that they could be migrants or hybrids between the two populations.

5.2.2 Isfjorden

Despite being geographically closer to several samples from the East Atlantic, the sample from Isfjorden in Svalbard clustered closely with samples from western Greenland. As this sample was highly divergent from off-shore Svalbard samples, and we did not detect any individuals with similar genetic make-up in any nearby samples, this fjord population may represent a regionally divergent gene pool, perhaps specially adapted to its Arctic environment [92]. Svalbard is surrounded by strong Arctic currents that run north and west towards the Arctic Ocean and the Greenland Sea (Supplementary Figure 6). However, the closest sample in Greenland in this study was over 3,000 kilometers away (along the shortest ice-free waterway). It is unknown how far north lumpfish reach, but historical Arctic connectivity could explain the observed pattern. However, pairwise F_{ST} estimates and Popgraph analysis show a higher resemblance between Isfjorden and southern Greenland compared to northern Greenland, suggesting a more recent connection. Either Isfjorden is an isolated relic of past gene-flow or a manifestation of still on-going connectivity. Strong cold polar currents have also been proposed as the barrier separating the West and East Atlantic

[43], Greenland and Canada, as well as Greenland and Iceland lumpfish populations [44]. These divisions align with our results, which showed significant divergence and no likely migrants across these borders.

5.2.3 Iceland

We found a weak but statistically significant east-west subdivision across the Westfjords in Iceland. Breiðafjörður appeared most distinct, whereas fish from Sandgerði and Bolungarvik were more admixed (Figure 4). This pattern was driven by only three outlier loci (Supplementary Table 2c), all of them in chromosome 13, and thus possibly suggesting a structural variant or a genomic region for local selection. A region in this chromosome, close to the outliers reported here, was recently discovered to be highly associated with sex [93], indicating that the observed structure could be related to differences between males and females. Repeated sampling of fish of opposite sex from the same location, Skagastrønd (SKA1_IS and SKA2_IS), did not show any significant differentiation, however (F_{ST} = 0.0007, NS). This suggests that sex is unlikely to bias the results.

5.2.4 English Channel

In the English Channel, lumpfish were genetically differentiated from all other samples (Table 2). This pattern was also observed using microsatellites in a previous study by Whittaker et al. (2018), which used the same individuals as in this study. As suggested therein, we agree that a plausible mechanism behind the southernmost population differentiation is warmer water (Supplementary Table 1a), causing separation in spawning time and thus temporal isolation restricting gene-flow.

5.2.5 Baltic Sea and the transition zone Skagerrak and Kattegat

We confirm the previous observations [43, 45] of high genetic divergence of the Baltic Sea lumpfish and high similarity between individuals within the Baltic (Figure 4; Supplementary Table 4a) and lower genome-wide variability (Supplementary Table 3). This is likely a combination of genetic drift due to limited number of founders and isolation, as well as strong diverging selection pressures in this marginal brackish environment, as has been shown for other species [17, 94, 95]. In fact, we show that variation in salinity together with temperature range, as well as minimum temperature with dissolved oxygen level, were significant factors explaining the genetic population structure of lumpfish. This was true even when the geographic variation was accounted for (Supplementary Figure 5), indicative of local adaptation in Baltic lumpfish. Nonetheless, one individual of Baltic Sea genetic origin was found in the Skagerrak off-shore sample, despite the large difference in salinity between the two areas, suggesting that adult Baltic Sea lumpfish can survive in a much more saline environment than they inhabit.

The salinity transition zone between the North Sea and Baltic Sea showed surprisingly high levels of structuring. While the Skagerrak area, adjacent to the North Sea, was clearly clustered with the larger East Atlantic group (see below), the Kattegat formed its own genetic cluster. Individuals from Orust in the northernmost parts of Kattegat showed some degree of assignment to the Skagerrak cluster. However, all other samples in Kattegat, including samples next to the entrance of the Baltic Sea, were highly differentiated from both the Baltic Sea and Skagerrak (Figure 4). A similar pattern has previously been discovered in other widespread marine fish [17, 96, 97], implying the strong environmental gradient here could support very localized population structures.

5.2.6 East Atlantic Group

Several geographically distant samples from the Barents Sea, Northern and Southern Norway, Skagerrak, North Sea and the Outer Hebrides formed a large admixed East Atlantic group. The East Atlantic group showed weak but clear north-south genetic differences in the clustering analysis (Supplementary Table 5). Furthermore, several samples appeared to include individuals of different genetic backgrounds, suggesting existence of cryptic structures. This applied especially to the southwestern Norwegian fjords, where about half of the fish clustered with the East Atlantic group. Similar mixtures of fish with different genetic backgrounds were also detected in the Arctic including the Barents Sea, and to a lesser extent Svalbard, Namsen, and Alta. The majority of these samples were migrating lumpfish of unknown origin. Based on our results, we suggest that at least two lumpfish populations are mechanically mixed in the Arctic waters, and that the Arctic group could breed in the coastal areas close to the Barents Sea from where we did not have breeding fish available. Alternatively, the genetic division could have arisen from different breeding times or from assortative mating [98]. Similar cryptic genetic subdivisions have recently been described for northern shrimp (*Pandalus borealis* [99]) and polar cod (*Boreogadus saida* [100]. We ruled out the inherent weakness of clustering analyses favoring solution K =2 [101, 102] as a plausible explanation for the observed two-cluster model in the Arctic, as well as for mainland Norway (see below) for several reasons: First, groupings were far from equal in size nor were they randomly distributed but constantly found within certain samples. Moreover, in the samples with several genetic groups, inbreeding coefficients were consistently higher than elsewhere (Supplementary Table 3), consistent with a Wahlund effect due to population subdivision.

It is still unclear if this large admixed East Atlantic group truly represents a single panmictic population – as previously suggested [45,46, 103]– or if it is simply a group of mechanically mixed individuals outside their breeding season. Even though these samples are more connected than suggested by their spatial distance (Supplementary Table 6), our data supported some internal subdivision within the group (Supplementary Table 5; see results for hierarchical clustering for “Similar East Atlantic sites”). As STRUCTURE estimates both ancestral gene frequencies and admixture proportions for each individual in relation to other samples, interpretation of weakly differing admixture proportions can easily be misleading, and over-interpretation should be avoided [104].

5.2.7 Norway

Along the coast of Norway, we discovered two main groups: fish from large fjords in southwestern Norway; Sognefjord, Hardangerfjord and Boknafjord (Supplementary Table 5), and fish which clustered with the large East Atlantic group described above (Alta, Flatanger and Flekkefjord). The southwestern fjords had two highly differentiated genetic clusters, where all individuals were clearly assigned to one genetic group or another. We had detailed catch information of all fish from one of the fjords, Boknafjord (data not shown). Examination of the genetic clusters did not reveal any relationship with the status of the fish (juvenile vs adult) nor sex but when we compared the genetic groups with the geographic location of each fish, spatial division was evident. Within this large fjord, ~100 km in length and covering an area of 1579 km^2, fish that were caught closer to the open sea clustered with the large East Atlantic group, whereas the group found mainly deeper inside the fjord formed another, distinct regional group (Supplementary Figure 7). This is likely the same genetic group as Whittaker et al. [45] reported, who also found a distinct genetic group from this same region.

It is unclear what the regionally diverged coastal group in the southwestern fjords represents, but there are some possibilities: First, these fish could represent local lumpfish ecotypes. Fjord ecotypes have been described for other fish species such as Atlantic cod [105], and are likely upheld by innate differences in feeding and movement ecologies [106]. The ecotype hypothesis is supported by a recent field observation from Hardangerfjord, where lumpfish of all sizes were recorded during the breeding time when fish of intermediate sizes are not expected (C. Durif, personal observation). Secondly, it is also possible that this diverged group originates from another colonization event than the East Atlantic group. A third possibility is that this unique genetic group could be of farmed origin. Even being of wild origin, farmed lumpfish are mass-produced from a small number of broodfish. Relatedness would skew population allele frequencies and could create distinct groupings [107, 108]. These large southwestern fjords are the central salmon farming area in Norway, and escaped farmed lumpfish there is possible and even likely [109]. However, a scenario where farmed fish of one distinct genotype would appear in such high numbers in several adjacent fjords simultaneously is unlikely. Broodstocks are primarily sourced from wild populations and thus unlikely to be this inbred.

5.3 Two datasets – pitfalls and advantages

Targeting outlier loci that often reflect divergent selection processes can offer a powerful way to study species with large population sizes, high dispersal capabilities and frequent gene-flow leading to population homogenization [110-112] and has proven to be a highly effective approach in cases where random sets of markers show little or no structure [113-115]. Our two-step approach enabled a comparison of two types of datasets to resolve population structuring for lumpfish. The genome-wide dataset allowed us to detect outlier loci putatively indicative of local adaptation and simultaneously identify a smaller cost-effective set of loci

to be genotyped in a large number of individuals with high geographic coverage [116]. The samples used in the initial 2b-RAD-analysis (see Table 1) and in the SNP selection phase were determined based on their location and previous knowledge of the genetic population structure for this species. This selection process has a few potential pitfalls: Studies conducted with a limited representation of the species' genome and based on few individuals can be prone to biases. The same applies to all later sampling stages if the samples used deviate from their source population (see e.g. [117-119]). Because genetic diversity is unequally distributed across populations, the populations in which SNPs are discovered may contribute to ascertainment bias. We tried to minimize known sources of biases in the marker development phase by including several sampling locations, using fish of both sexes and selecting markers with a wide genomic coverage. Whenever possible, samples from each site were collected at multiple time points and from a wider area. As RADseq is a non-selective process in relation to which genomic regions are to be included, the final genomic representation is random. Thus, population processes mediated by neutral genetic markers, such as demographic history, can reliably be studied as they usually affect the entire genome. However, selection-related processes can be missed since selection often affects relatively small and targeted parts of the genome, many of which may not be included in random RADseqs [120]. Therefore, this study will tell only a partial and likely somewhat biased story of the lumpfish population structure, and more comprehensive studies – geographically and/or genome-wise – could provide with other pieces of evidence.

However, outlier analysis remains a powerful approach to study larger patterns of differentiation. In this study, we found that the targeted dataset performed equally well or even better than the genome-wide dataset at separating lumpfish populations regionally. This is despite the fact that only a small portion (3-17 SNPs; Supplementary Table 2c) of the

selected 139 SNPs were informative within each region. The targeted dataset displayed ~2 to 5 times higher level of divergence (Table 2), and greater clarity than the genome-wide dataset (Figure 3). In addition to the previously described population structure, our analyses revealed further genetic structure. Furthermore, our analyses revealed evidence of likely migrants and hybrids on both sides of the Atlantic.

5.4 Conclusions and management implications

Lumpfish occupy vast and highly variable marine environments and have high migration ability. However, based on its trophic level and long estimated population doubling time, lumpfish is expected to have low resilience to fishing pressure [121]. The impact of past and present fisheries on populations of lumpfish is largely unknown, and likely to be highly varying among regions and countries due to differences in management [25]. Moreover, the net productivity of lumpfish stocks may vary considerably between regions [122]. Significant declines in stock abundances have been reported in Canada, where lumpfish was designated as threatened in 2017 [123], whereas Norwegian and Barents Sea lumpfish stocks have lately been relatively stable or even increasing [124-126]. Our results show that lumpfish is a highly-structured species with local stocks which need to be managed as such although the stocks may mix physically outside of spawning season. Lumpfish is mainly targeted during the spawning season, which reduces the risk related to fisheries targeting mixed stocks [127]. Nevertheless, overfishing could still be a problem as there is likely little migration between spawning areas and repopulating overfished stocks could take a long time.

The use of lumpfish as a cleaner fish in commercial aquaculture has shown a rapid increase during the last decade, quickly becoming the most commonly used cleaner fish species for salmon farming [128]. Even though most lumpfish utilized in salmon farms are of farmed

origin, broodstocks are still primarily sourced from wild fish. Currently, there are no regulations regarding the origin of cleaner fish released into the net-pens along the vast Norwegian coastline [128]. Although, there is evidence of lumpfish being shipped and translocated, the degree is not known [129]. Translocating fish can have many consequences for local populations, species and even ecosystems. Introduced organisms can bring foreign diseases and parasites novel to local ecosystems [130]. If the introduced organisms are genetically divergent from local populations, they may introduce unfavorable genetic material, which can result in altered population subdivision [30], reduced genetic variation, and/or reduced fitness [29, 131]. Local adaptation, together with the observed strong, clear regional population subdivision likely upheld by natal fidelity, emphasizes the need to manage the species on a regional level despite its wide transatlantic distribution. Considering on-going climate change for cold-adapted species like lumpfish, the standing genetic variation, especially in the southernmost latitudes of the species range might prove to be an invaluable resource in the future. The results from this study improve upon existing knowledge of lumpfish populations, which should be taken into consideration for the future use and translocation of lumpfish to be used in the aquaculture industry. This study also provides a baseline for future monitoring of wild populations. The panel of SNPs used in this study has a high power to distinguish lumpfish from different populations and could in the future be applied for identifying wild and translocated fish.

6 Acknowledgements

We are grateful to those many people who helped us acquiring and handling the samples used in this study: Eva Farestveit, Elizabeth A. Fairchild, Vidar Wennevik, Gunnar Bakke, Lucilla Giulietti, John Fredrik Strøm, Jon-Ivar Westgaard, Ole Ingar Paulsen, Fredrik Staven, Håkan Wennhage, Trond Rafoss, Lauri Pietikäinen, Johanne Gauthier, Kim Aarestrup, Kim Birnie-

Gauvin, Carlos García de Leániz, Sofía Consuegra, Rasmus Hedeholm, Maia Olsen, Carina Jernberg, Alain Fréchet, Sally Sherman, staff at the Centre for Sustainable Aquatic Research and the personnel on research vessels *Johan Hjort* and *G.O. Sars.* 2bRAD sequencing was performed by the SNP&SEQ Technology Platform in Uppsala. The facility is part of the National Genomics Infrastructure (NGI) Sweden and Science for Life Laboratory. The SNP&SEQ Platform is also supported by the Swedish Research Council and the Knut and Alice Wallenberg Foundation. The work was performed at the Institute of Marine Research, Bergen Norway (www.hi.no) and within the Linnaeus Centre for Marine Evolutionary Biology (www.cemeb.science.gu.se/). Earlier versions of this manuscript have previously been published in a thesis [132] and as a preprint in bioRxiv [133].

7 References

1 FAO. The State of World Fisheries and Aquaculture 2018 - Meeting the sustainable development goals. Rome: 2018.

2 Allendorf FW, England PR, Luikart G, Ritchie PA, Ryman N. Genetic effects of harvest on wild animal populations. Trends Ecol Evol. 2008;23(6):327-37. Epub 2008/04/29. doi: 10.1016/j.tree.2008.02.008. PubMed PMID: 18439706.

3 Lowe WH, Allendorf FW. What can genetics tell us about population connectivity? Mol Ecol. 2010;19(15):3038-51. Epub 2010/07/14. doi: 10.1111/j.1365-294X.2010.04688.x. PubMed PMID: 20618697.

4 Lowe WH, Kovach RP, Allendorf FW. Population Genetics and Demography Unite Ecology and Evolution. Trends Ecol Evol. 2017;32(2):141-52. Epub 2017/01/17. doi: 10.1016/j.tree.2016.12.002. PubMed PMID: 28089120.

5 Waples RS, Punt AE, Cope JM. Integrating genetic data into management of marine resources: how can we do it better? Fish and Fisheries. 2008;9:423-49.

6 Reiss H, Hoarau G, Dickey-Collas M, Wolff WJ. Genetic population structure of marine fish: mismatch between biological and fisheries management units. Fish and Fisheries. 2009;10(4):361-95. doi: 10.1111/j.1467-2979.2008.00324.x.

7 Pinsky ML, Palumbi SR. Meta-analysis reveals lower genetic diversity in overfished populations. Mol Ecol. 2014;23(1):29-39. Epub 2014/01/01. doi: 10.1111/mec.12509. PubMed PMID: 24372754.

8 Waples RS, Gaggiotti O. What is a population? An empirical evaluation of some genetic methods for identifying the number of gene pools and their degree of connectivity. Mol Ecol. 2006;15(6):1419-39. Epub 2006/04/25. doi: 10.1111/j.1365-294X.2006.02890.x. PubMed PMID: 16629801.

9 Portnoy DS, McDowell JR, McCandless CT, Musick JA, Graves JE. Effective size closely approximates the census size in the heavily exploited western Atlantic population of the sandbar

shark, Carcharhinus plumbeus. Conservation Genetics. 2008;10(6):1697-705. doi: 10.1007/s10592-008-9771-2.

10 Palstra FP, Ruzzante DE. Genetic estimates of contemporary effective population size: what can they tell us about the importance of genetic stochasticity for wild population persistence? Mol Ecol. 2008;17(15):3428-47. Epub 2009/01/23. doi: 10.1111/j.1365-294x.2008.03842.x. PubMed PMID: 19160474.

11 Spies I, Hauser L, Jorde PE, Knutsen H, Punt AE, Rogers LA, et al. Inferring genetic connectivity in real populations, exemplified by coastal and oceanic Atlantic cod. Proc Natl Acad Sci U S A. 2018;115(19):4945-50. Epub 2018/04/21. doi: 10.1073/pnas.1800096115. PubMed PMID: 29674450; PubMed Central PMCID: PMCPMC5948993.

12 Van Wyngaarden M, Snelgrove PV, DiBacco C, Hamilton LC, Rodriguez-Ezpeleta N, Jeffery NW, et al. Identifying patterns of dispersal, connectivity and selection in the sea scallop, Placopecten magellanicus, using RADseq-derived SNPs. Evol Appl. 2017;10(1):102-17. Epub 2016/12/31. doi: 10.1111/eva.12432. PubMed PMID: 28035239; PubMed Central PMCID: PMCPMC5192885.

13 Gonçalves da Silva A, Barendse W, Kijas J, England PR, Hoelzel AR, Coleman M. Genomic data suggest environmental drivers of fish population structure in the deep sea: A case study for the orange roughy (Hoplostethus atlanticus). Journal of Applied Ecology. 2019;57(2):296-306. doi: 10.1111/1365-2664.13534.

14 Palumbi SR, Evans TG, Pespeni MH, Somero GN. Present and Future Adaptation of Marine Species Assemblages: DNA-Based Insights into Climate Change from Studies of Physiology, Genomics, and Evolution. Oceanography. 2019;32(3):82-93. doi: 10.5670/oceanog.2019.314.

15 Han F, Jamsandekar M, Pettersson ME, Su L, Fuentes-Pardo AP, Davis BW, et al. Ecological adaptation in Atlantic herring is associated with large shifts in allele frequencies at hundreds of loci. Elife. 2020;9. Epub 2020/12/05. doi: 10.7554/eLife.61076. PubMed PMID: 33274714; PubMed Central PMCID: PMCPMC7738190.

16 Kerr LA, Hintzen NT, Cadrin SX, Clausen LW, Dickey-Collas M, Goethel DR, et al. Lessons learned from practical approaches to reconcile mismatches between biological population structure and stock units of marine fish. ICES Journal of Marine Science. 2017;74(6):1708-22. doi: 10.1093/icesjms/fsw188.

17 Quintela M, Kvamme C, Bekkevold D, Nash RDM, Jansson E, Sorvik AG, et al. Genetic analysis redraws the management boundaries for the European sprat. Evol Appl. 2020;13(8):1906-22. Epub 2020/09/11. doi: 10.1111/eva.12942. PubMed PMID: 32908594; PubMed Central PMCID: PMCPMC7463317.

18 Hemmer-Hansen J, Hussy K, Baktoft H, Huwer B, Bekkevold D, Haslob H, et al. Genetic analyses reveal complex dynamics within a marine fish management area. Evol Appl. 2019;12(4):830-44. Epub 2019/04/13. doi: 10.1111/eva.12760. PubMed PMID: 30976313; PubMed Central PMCID: PMCPMC6439499.

19 Huret M, Lebigre C, Iriondo M, Montes I, Estonba A. Genetic population structure of anchovy (Engraulis encrasicolus) in North-western Europe and variability in the seasonal distribution of the stocks. Fisheries Research. 2020;229. doi: 10.1016/j.fishres.2020.105619.

20 Maroso F, Gkagkavouzis K, De Innocentiis S, Hillen J, do Prado F, Karaiskou N, et al. Genome-wide analysis clarifies the population genetic structure of wild Gilthead Sea Bream (Sparus aurata). bioRxiv preprint2020.

21 Xuereb A, D'Aloia CC, Andrello M, Bernatchez L, Fortin MJ. Incorporating putatively neutral and adaptive genomic data into marine conservation planning. Conserv Biol. 2020. Epub 2020/08/14. doi: 10.1111/cobi.13609. PubMed PMID: 32785955.

22 Hohenlohe PA, Funk WC, Rajora OP. Population genomics for wildlife conservation and management. Mol Ecol. 2021;30(1):62-82. Epub 2020/11/05. doi: 10.1111/mec.15720. PubMed PMID: 33145846; PubMed Central PMCID: PMCPMC7894518.

23 Hvas M, Folkedal O, Imsland A, Oppedal F. Metabolic rates, swimming capabilities, thermal niche and stress response of the lumpfish, Cyclopterus lumpus. Biol Open. 2018;7(9). Epub 2018/08/18. doi: 10.1242/bio.036079. PubMed PMID: 30115616; PubMed Central PMCID: PMCPMC6176939.

24 Davenport J. Synopsis of biological data on the lumpsucker Cycloptrus lunzpus (Linnaeus, 1758). Food and agriculture organization of the United Nations, 1985 147.

25 Kennedy J, Durif CMF, Florin A-B, Fréchet A, Gauthier J, Hüssy K, et al. A brief history of lumpfishing, assessment, and management across the North Atlantic. ICES Journal of Marine Science. 2019;76(1):181-91. doi: 10.1093/icesjms/fsy146.

26 Imsland AKD, Reynolds P, Eliassen G, Hangstad TA, Foss A, Vikingstad E, et al. The use of lumpfish (Cyclopterus lumpus L.) to control sea lice (Lepeophtheirus salmonis Krøyer) infestations in intensively farmed Atlantic salmon (Salmo salar L.). Aquaculture. 2014;424-425:18-23. doi: 10.1016/j.aquaculture.2013.12.033.

27 Imsland AKD, Hanssen A, Nytro AV, Reynolds P, Jonassen TM, Hangstad TA, et al. It works! Lumpfish can significantly lower sea lice infestation in large-scale salmon farming. Biol Open. 2018;7(9). Epub 2018/09/05. doi: 10.1242/bio.036301. PubMed PMID: 30177547; PubMed Central PMCID: PMCPMC6176945.

28 Powell A, Treasurer JW, Pooley CL, Keay AJ, Lloyd R, Imsland AK, et al. Use of lumpfish for sea-lice control in salmon farming: challenges and opportunities. Reviews in Aquaculture. 2018;10(3):683-702. doi: 10.1111/raq.12194.

29 Laikre L, Schwartz MK, Waples RS, Ryman N, GeM Working Group. Compromising genetic diversity in the wild: unmonitored large-scale release of plants and animals. Trends Ecol Evol. 2010;25(9):520-9. Epub 2010/08/07. doi: 10.1016/j.tree.2010.06.013. PubMed PMID: 20688414.

30 Glover KA, Quintela M, Wennevik V, Besnier F, Sorvik AG, Skaala O. Three decades of farmed escapees in the wild: a spatio-temporal analysis of Atlantic salmon population genetic structure throughout Norway. PLoS One. 2012;7(8):e43129. Epub 2012/08/24. doi: 10.1371/journal.pone.0043129. PubMed PMID: 22916215; PubMed Central PMCID: PMCPMC3419752.

31 Wringe BF, Jeffery NW, Stanley RRE, Hamilton LC, Anderson EC, Fleming IA, et al. Extensive hybridization following a large escape of domesticated Atlantic salmon in the Northwest Atlantic. Commun Biol. 2018;1:108. Epub 2018/10/03. doi: 10.1038/s42003-018-0112-9. PubMed PMID: 30271988; PubMed Central PMCID: PMCPMC6123692.

32 Jansson E, Quintela M, Dahle G, Albretsen J, Knutsen H, André C, et al. Genetic analysis of goldsinny wrasse reveals evolutionary insights into population connectivity and potential evidence of inadvertent translocation via aquaculture. ICES Journal of Marine Science. 2017;74(8):2135-47. doi: 10.1093/icesjms/fsx046.

33 Faust E, Halvorsen KT, Andersen P, Knutsen H, Andre C. Cleaner fish escape salmon farms and hybridize with local wrasse populations. R Soc Open Sci. 2018;5(3):171752. Epub 2018/04/17. doi: 10.1098/rsos.171752. PubMed PMID: 29657779; PubMed Central PMCID: PMCPMC5882703.

34 Faust E, Jansson E, André C, Halvorsen KT, Dahle G, Knutsen H, et al. Not that clean: Aquaculture- mediated translocation of cleaner fish has led to hybridization on the northern edge of the species' range. Evolutionary Applications. 2021. doi: 10.1111/eva.13220.

35 Nøttestad L, Anthonypillai V, dos Santos Schmidt TC, Høines Å, Salthaug A, Ólafsdóttir AH, et al. Cruise report from the International Ecosystem Summer Survey in the Nordic Seas (IESSNS).

Working doucment for ICES Working Group on Widely Distributed Stocks (WGWIDE, No. 5). 2020.

36 Kennedy J, Jónsson SÞ, Ólafsson HG, Kasper JM. Observations of vertical movements and depth distribution of migrating female lumpfish (Cyclopterus lumpus) in Iceland from data storage tags and trawl surveys. ICES Journal of Marine Science. 2016;73(4):1160-9. doi: 10.1093/icesjms/fsv244.

37 Mitamura H, Thorstad EB, Uglem I, Bjørn PA, Økland F, Næsje TF, et al. Movements of lumpsucker females in a northern Norwegian fjord during the spawning season. Environmental Biology of Fishes. 2012;93(4):475-81. doi: 10.1007/s10641-011-9942-8.

38 Ingólfsson A. Colonization of floating seaweed by pelagic and subtidal benthic animals in southwestern Iceland. Hydrobiologia. 2000;440:181-9.

39 Kasper JM, Bertelsen B, Ólafsson HG, Holst JC, Sturlaugsson J, Jónsson SP. Observations of growth and postspawning survival of lumpfish Cyclopterus lumpus from mark-recapture studies. 2014;84(6):1958-63. doi: 10.1111/jfb.12389.

40 Hedeholm RB, Blicher ME, Grønkjær P. First estimates of age and production of lumpsucker (Cyclopterus lumpus) in Greenland. Fisheries Research. 2014;149:1-4. doi: 10.1016/j.fishres.2013.08.016.

41 Kennedy J, Ólafsson HG. Conservation of spawning time between years in lumpfish Cyclopterus lumpus and potential impacts from the temporal distribution of fishing effort. 2019;26(4):389-96. doi: 10.1111/fme.12352.

42 Kennedy J, Jónsson SÞ, Kasper JM, Ólafsson HG. Movements of female lumpfish (Cyclopterus lumpus) around Iceland. ICES Journal of Marine Science. 2015;72(3):880-9. doi: 10.1093/icesjms/fsu170.

43 Pampoulie C, Skirnisdottir S, Olafsdottir G, Helyar SJ, Thorsteinsson V, Jónsson SÞ, et al. Genetic structure of the lumpfish Cyclopterus lumpus across the North Atlantic. ICES Journal of Marine Science. 2014;71(9):2390-7. doi: 10.1093/icesjms/fsu071.

44 Garcia-Mayoral E, Olsen M, Hedeholm R, Post S, Nielsen EE, Bekkevold D. Genetic structure of West Greenland populations of lumpfish Cyclopterus lumpus. J Fish Biol. 2016;89(6):2625-42. Epub 2016/10/19. doi: 10.1111/jfb.13167. PubMed PMID: 27753091.

45 Whittaker BA, Consuegra S, Garcia de Leaniz C. Genetic and phenotypic differentiation of lumpfish (Cyclopterus lumpus) across the North Atlantic: implications for conservation and aquaculture. PeerJ. 2018;6:e5974. Epub 2018/12/01. doi: 10.7717/peerj.5974. PubMed PMID: 30498640; PubMed Central PMCID: PMCPMC6251346.

46 Jónsdóttir ÓDB, Schregel J, Hagen SB, Tobiassen C, Aarnes SG, Imsland AKD. Population genetic structure of lumpfish along the Norwegian coast: aquaculture implications. Aquaculture International. 2017;26(1):49-60. doi: 10.1007/s10499-017-0194-2.

47 Davey JW, Blaxter ML. RADSeq: next-generation population genetics. Brief Funct Genomics. 2010;9(5-6):416-23. Epub 2011/01/27. doi: 10.1093/bfgp/elq031. PubMed PMID: 21266344; PubMed Central PMCID: PMCPMC3080771.

48 Pante E, Simon-Bouhet B. marmap: A Package for Importing, Plotting and Analyzing Bathymetric and Topographic Data in R. PLoS ONE. 2013;8(9)(e73051). doi:10.1371/journal.pone.0073051.

49 R Core Team. R: A language and environment for statistical computing. R Foundation for Statistical Computing, Vienna, Austria. URL https://www.R-project.org/. 2020.

50 Wang S, Meyer E, McKay JK, Matz MV. 2b-RAD: a simple and flexible method for genome-wide genotyping. Nature Methods. 2012;9:808-10. doi: 10.1038/nmeth.2023.

51 Knutsen TM, Kirubakaran GT, Mommens M, Moen T. Lumpfish (Cyclopterus lumpus) draft genome assembly. figshare. Database:figshare [Internet]. Available from: https://doi.org/10.6084/m9.figshare.7301546.v4

52 Langmead B, Salzberg SL. Fast gapped-read alignment with Bowtie 2. Nat Methods. 2012;9(4):357-9. Epub 2012/03/06. doi: 10.1038/nmeth.1923. PubMed PMID: 22388286; PubMed Central PMCID: PMCPMC3322381.

53 McKenna A, Hanna M, Banks E, Sivachenko A, Cibulskis K, Kernytsky A, et al. The Genome Analysis Toolkit: a MapReduce framework for analyzing next-generation DNA sequencing data. Genome Res. 2010;20(9):1297-303. Epub 2010/07/21. doi: 10.1101/gr.107524.110. PubMed PMID: 20644199; PubMed Central PMCID: PMCPMC2928508.

54 Keenan K, McGinnity P, Cross TF, Crozier WW, Prodöhl PA, O'Hara RB. diveRsity: AnRpackage for the estimation and exploration of population genetics parameters and their associated errors. Methods in Ecology and Evolution. 2013;4(8):782-8. doi: 10.1111/2041-210x.12067.

55 Gabriel S, Ziaugra L, Tabbaa D. SNP genotyping using the Sequenom MassARRAY iPLEX platform. Curr Protoc Hum Genet. 2009;Chapter 2:Unit 2 12. Epub 2009/01/27. doi: 10.1002/0471142905.hg0212s60. PubMed PMID: 19170031.

56 Gruber B, Unmack PJ, Berry OF, Georges A. dartr: An r package to facilitate analysis of SNP data generated from reduced representation genome sequencing. Mol Ecol Resour. 2018;18(3):691-9. Epub 2017/12/22. doi: 10.1111/1755-0998.12745. PubMed PMID: 29266847.

57 Rousset F. genepop'007: a complete re-implementation of the genepop software for Windows and Linux. Mol Ecol Resour. 2008;8(1):103-6. Epub 2008/01/01. doi: 10.1111/j.1471-8286.2007.01931.x. PubMed PMID: 21585727.

58 Kamvar ZN, Tabima JF, Grunwald NJ. Poppr: an R package for genetic analysis of populations with clonal, partially clonal, and/or sexual reproduction. PeerJ. 2014;2:e281. Epub 2014/04/02. doi: 10.7717/peerj.281. PubMed PMID: 24688859; PubMed Central PMCID: PMCPMC3961149.

59 Dray S, Dufour A-B. The ade4 Package: Implementing the Duality Diagram for Ecologists. Journal of Statistical Software. 2007;22(4).

60 Weir BS, Cockerham CC. Estimating F-Statistics for the Analysis of Population Structure. Evolution. 1984;38(6). doi: 10.2307/2408641.

61 Pembleton LW, Cogan NO, Forster JW. StAMPP: an R package for calculation of genetic differentiation and structure of mixed-ploidy level populations. Mol Ecol Resour. 2013;13(5):946-52. Epub 2013/06/07. doi: 10.1111/1755-0998.12129. PubMed PMID: 23738873.

62 Benjamini Y, Hochberg Y. Controlling the False Discovery Rate: A Practical and Powerful Approach to Multiple Testing. Journal of the Royal Statistical Society: Series B. 1995;57(1):289-300.

63 Chessel D, Dufour A-B, Thioulouse J. The ade4 package - I : One-table methods. R News. 2004;7(2):47-52.

64 Jombart T. adegenet: a R package for the multivariate analysis of genetic markers. Bioinformatics. 2008;24(11):1403-5. Epub 2008/04/10. doi: 10.1093/bioinformatics/btn129. PubMed PMID: 18397895.

65 Jombart T, Ahmed I. adegenet 1.3-1: new tools for the analysis of genome-wide SNP data. Bioinformatics. 2011;27(21):3070-1. Epub 2011/09/20. doi: 10.1093/bioinformatics/btr521. PubMed PMID: 21926124; PubMed Central PMCID: PMCPMC3198581.

66 Pritchard JK, Stephens M, Donnelly P. Inference of Population Structure Using Multilocus Genotype Data. Genetics. 2000;155:945-59.

67 Falush D, Stephens M, Pritchard JK. Inference of Population Structure Using Multilocus Genotype Data: Linked Loci and Correlated Allele Frequencies. Genetics. 2003;164:1567-87.

68 Hubisz MJ, Falush D, Stephens M, Pritchard JK. Inferring weak population structure with the assistance of sample group information. Mol Ecol Resour. 2009;9(5):1322-32. Epub 2009/09/01. doi: 10.1111/j.1755-0998.2009.02591.x. PubMed PMID: 21564903; PubMed Central PMCID: PMCPMC3518025.

69 Li YL, Liu JX. StructureSelector: A web-based software to select and visualize the optimal number of clusters using multiple methods. Mol Ecol Resour. 2018;18(1):176-7. Epub 2017/09/19. doi: 10.1111/1755-0998.12719. PubMed PMID: 28921901.

70 Evanno G, Regnaut S, Goudet J. Detecting the number of clusters of individuals using the software STRUCTURE: a simulation study. Mol Ecol. 2005;14(8):2611-20. Epub 2005/06/23. doi: 10.1111/j.1365-294X.2005.02553.x. PubMed PMID: 15969739.

71 Puechmaille SJ. The program structure does not reliably recover the correct population structure when sampling is uneven: subsampling and new estimators alleviate the problem. Mol Ecol Resour. 2016;16(3):608-27. Epub 2016/02/10. doi: 10.1111/1755-0998.12512. PubMed PMID: 26856252.

72 Kopelman NM, Mayzel J, Jakobsson M, Rosenberg NA, Mayrose I. Clumpak: a program for identifying clustering modes and packaging population structure inferences across K. Mol Ecol Resour. 2015;15(5):1179-91. Epub 2015/02/17. doi: 10.1111/1755-0998.12387. PubMed PMID: 25684545; PubMed Central PMCID: PMCPMC4534335.

73 Dyer RJ, Nason JD. Population Graphs: the graph theoretic shape of genetic structure. Molecular Ecology. 2004;13(7):1713-27.

74 Dyer RJ, Nason JD, Garrick RC. Landscape modelling of gene flow: improved power using conditional genetic distance derived from the topology of population networks. Molecular Ecology. 2010;19(17):3746-59. doi: 10.1111/j.1365-294X.2010.04748.x.

75 Dyer RJ. Package ‘gstudio’: tools related to the spatial analysis of genetic marker data, R package version 1.3. 2015.

76 Dyer RJ. Package ‘popgraph’: an R package that constructs and manipulates population graphs. 1.4 ed2015.

77 Csardi G, Nepusz T. The igraph software package for complex network research. InterJournal. 2006;Complex Systems.

78 Tyberghein L, Verbruggen H, Pauly K, Troupin C, Mineur F, De Clerck O. Bio-ORACLE: a global environmental dataset for marine species distribution modelling. Global Ecology and Biogeography. 2012;21(2):272-81. doi: 10.1111/j.1466-8238.2011.00656.x.

79 Assis J, Tyberghein L, Bosch S, Verbruggen H, Serrão EA, De Clerck O. Bio-ORACLE v2.0: Extending marine data layers for bioclimatic modelling. Global Ecology and Biogeography. 2018;27(3):277-84. doi: 10.1111/geb.12693.

80 Kuhn M, Jackson S, Cimentada J. corrr: Correlations in R. R package version 0.4.3. https://CRAN.R-project.org/package=corrr. 2020.

81 Wei T, Simko V. R package "corrplot": Visualization of a Correlation Matrix (Version 0.84). Available from https://github.com/taiyun/corrplot. 2017.

82 Chavent M, Kuentz V, Liquet B, Saracco J. ClustOfVar: Clustering of Variables. R package version 1.1. https://CRAN.R-project.org/package=ClustOfVar. 2017.

83 Frichot E, François O. LEA: An R package for landscape and ecological association studies. Methods in Ecology and Evolution. 2015;6(8):925-9. doi: 10.1111/2041-210x.12382.

84 Storey JD, Bass AJ, Dabney A, Robinson D. qvalue: Q-value estimation for false discovery rate control. 2020.

85 Luu K, Bazin E, Blum MG. pcadapt: an R package to perform genome scans for selection based on principal component analysis. Mol Ecol Resour. 2017;17(1):67-77. Epub 2016/09/08. doi: 10.1111/1755-0998.12592. PubMed PMID: 27601374.

86 Capblancq T, Luu K, Blum MGB, Bazin E. Evaluation of redundancy analysis to identify signatures of local adaptation. Mol Ecol Resour. 2018;18(6):1223-33. Epub 2018/05/29. doi: 10.1111/1755-0998.12906. PubMed PMID: 29802785.

87 Dray S, Bauman D, Blanchet G, Borcard D, Clappe S, Guenard G, et al. adespatial: Multivariate Multiscale Spatial Analysis. R package version 0.3-14. https://CRAN.R-project.org/package=adespatial. 2021.

88 Oksanen J, Blanchet FG, Friendly M, Kindt R, Legendre P, McGlinn D, et al. vegan: Community Ecology Package. R package version 2.5-7. https://CRAN.R-project.org/package=vegan. 2020.

89 Bonanomi S, Overgaard Therkildsen N, Retzel A, Berg Hedeholm R, Pedersen MW, Meldrup D, et al. Historical DNA documents long-distance natal homing in marine fish. Mol Ecol. 2016;25(12):2727-34. Epub 2016/02/10. doi: 10.1111/mec.13580. PubMed PMID: 26859133.

90 Carr SM, Marshall HD. Intraspecific phylogeographic genomics from multiple complete mtDNA genomes in Atlantic cod (Gadus morhua): origins of the "codmother," transatlantic vicariance and midglacial population expansion. Genetics. 2008;180(1):381-9. Epub 2008/08/22. doi: 10.1534/genetics.108.089730. PubMed PMID: 18716332; PubMed Central PMCID: PMCPMC2535689.

91 Li J-J, Hu Z-M, Duan D-L, Rocha L. Genetic data from the red alga Palmaria palmata reveal a mid-Pleistocene deep genetic split in the North Atlantic. Journal of Biogeography. 2015;42(5):902-13. doi: 10.1111/jbi.12464.

92 Skogseth R, Olivier LLA, Nilsen F, Falck E, Fraser N, Tverberg V, et al. Variability and decadal trends in the Isfjorden (Svalbard) ocean climate and circulation – An indicator for climate change in the European Arctic. Progress in Oceanography. 2020;187. doi: 10.1016/j.pocean.2020.102394.

93 Holborn MK, Einfeldt AL, Kess T, Duffy SJ, Messmer AM, Langille BL, et al. Reference genome of Lumpfish Cyclopterus lumpus Linnaeus provides evidence of male heterogametic sex determination through the AMH pathway. Mol Ecol Resour. 2021. Epub 2021/12/04. doi: 10.1111/1755-0998.13565. PubMed PMID: 34859595.

94 Johannesson K, André C. Life on the margin: genetic isolation and diversity loss in a peripheral marine ecosystem, the Baltic Sea. Mol Ecol. 2006;15(8):2013-29. Epub 2006/06/20. doi: 10.1111/j.1365-294X.2006.02919.x. PubMed PMID: 16780421.

95 Guo B, Li Z, Merila J. Population genomic evidence for adaptive differentiation in the Baltic Sea herring. Mol Ecol. 2016;25(12):2833-52. Epub 2016/04/20. doi: 10.1111/mec.13657. PubMed PMID: 27093194.

96 Gaggiotti OE, Bekkevold D, Jorgensen HB, Foll M, Carvalho GR, Andre C, et al. Disentangling the effects of evolutionary, demographic, and environmental factors influencing genetic structure of natural populations: Atlantic herring as a case study. Evolution. 2009;63(11):2939-51. Epub 2009/07/25. doi: 10.1111/j.1558-5646.2009.00779.x. PubMed PMID: 19624724.

97 Johannesson K, Le Moan A, Perini S, Andre C. A Darwinian Laboratory of Multiple Contact Zones. Trends Ecol Evol. 2020. Epub 2020/09/12. doi: 10.1016/j.tree.2020.07.015. PubMed PMID: 32912631.

98 Potkamp G, Fransen CHJM. Speciation with gene flow in marine systems. Contributions to Zoology. 2019:1-40. doi: 10.1163/18759866-20191344.

99 Hansen A, Westgaard J-I, Søvik G, Hanebrekke T, Nilssen EM, Jorde PE, et al. Genetic differentiation between inshore and offshore populations of northern shrimp (Pandalus borealis). ICES Journal of Marine Science. 2021. doi: 10.1093/icesjms/fsab181.

100 Quintela M, Bhat S, Præbel K, Gordeeva N, Seljestad GW, Hanebrekke T, et al. Distinct genetic clustering in the weakly differentiated polar cod, Boreogadus saida Lepechin, 1774 from East Siberian Sea to Svalbard. Polar Biology. 2021;44(8):1711-24. doi: 10.1007/s00300-021-02911-7

101 Quintela M, Skaug HJ, Oien N, Haug T, Seliussen BB, Solvang HK, et al. Investigating population genetic structure in a highly mobile marine organism: the minke whale Balaenoptera acutorostrata acutorostrata in the North East Atlantic. PLoS One. 2014;9(9):e108640. Epub 2014/10/01. doi: 10.1371/journal.pone.0108640. PubMed PMID: 25268591; PubMed Central PMCID: PMCPMC4182549.

102 Janes JK, Miller JM, Dupuis JR, Malenfant RM, Gorrell JC, Cullingham CI, et al. The K = 2 conundrum. Mol Ecol. 2017;26(14):3594-602. Epub 2017/05/26. doi: 10.1111/mec.14187. PubMed PMID: 28544181.

103 Jónsdóttir ÓDB, Gíslason D, Ólafsdóttir G, Maduna S, Hagen SB, Reynolds P, et al. Lack of population genetic structure of lumpfish along the Norwegian coast: A reappraisal based on EST-STRs analyses. Aquaculture. 2022;555(738230). doi: 10.1016/j.aquaculture.2022.738230

104 Lawson DJ, van Dorp L, Falush D. A tutorial on how not to over-interpret STRUCTURE and ADMIXTURE bar plots. Nat Commun. 2018;9(1):3258. Epub 2018/08/16. doi: 10.1038/s41467-018-05257-7. PubMed PMID: 30108219; PubMed Central PMCID: PMCPMC6092366.

105 Knutsen H, Jorde PE, Hutchings JA, Hemmer-Hansen J, Gronkjaer P, Jorgensen KM, et al. Stable coexistence of genetically divergent Atlantic cod ecotypes at multiple spatial scales. Evol Appl. 2018;11(9):1527-39. Epub 2018/10/23. doi: 10.1111/eva.12640. PubMed PMID: 30344625; PubMed Central PMCID: PMCPMC6183466.

106 Kristensen ML, Olsen EM, Moland E, Knutsen H, Gronkjaer P, Koed A, et al. Disparate movement behavior and feeding ecology in sympatric ecotypes of Atlantic cod. Ecol Evol. 2021;11(16):11477-90. Epub 2021/08/26. doi: 10.1002/ece3.7939. PubMed PMID: 34429934; PubMed Central PMCID: PMCPMC8366838.

107 Glover KA, Dahle G, Jørstad KE. Genetic identification of farmed and wild Atlantic cod, Gadus morhua, in coastal Norway. ICES Journal of Marine Science. 2011;68(5):901-10. doi: 10.1093/icesjms/fsr048.

108 O'Connell KA, Mulder KP, Maldonado J, Currie KL, Ferraro DM. Sampling related individuals within ponds biases estimates of population structure in a pond-breeding amphibian. Ecol Evol. 2019;9(6):3620-36. Epub 2019/04/10. doi: 10.1002/ece3.4994. PubMed PMID: 30962914; PubMed Central PMCID: PMCPMC6434569.

109 Herrmann B, Sistiaga M, Jorgensen T. Size-dependent escape risk of lumpfish (Cyclopterus lumpus) from salmonid farm nets. Mar Pollut Bull. 2021;162:111904. Epub 2020/12/16. doi: 10.1016/j.marpolbul.2020.111904. PubMed PMID: 33321307.

110 Milano I, Babbucci M, Cariani A, Atanassova M, Bekkevold D, Carvalho GR, et al. Outlier SNP markers reveal fine-scale genetic structuring across European hake populations (Merluccius merluccius). Mol Ecol. 2014;23(1):118-35. Epub 2013/10/22. doi: 10.1111/mec.12568. PubMed PMID: 24138219.

111 Tigano A, Shultz AJ, Edwards SV, Robertson GJ, Friesen VL. Outlier analyses to test for local adaptation to breeding grounds in a migratory arctic seabird. Ecol Evol. 2017;7(7):2370-81. Epub 2017/04/14. doi: 10.1002/ece3.2819. PubMed PMID: 28405300; PubMed Central PMCID: PMCPMC5383466.

112 Jenkins TL, Ellis CD, Triantafyllidis A, Stevens JR. Single nucleotide polymorphisms reveal a genetic cline across the north-east Atlantic and enable powerful population assignment in the European lobster. Evol Appl. 2019;12(10):1881-99. Epub 2019/11/09. doi: 10.1111/eva.12849. PubMed PMID: 31700533; PubMed Central PMCID: PMCPMC6824076.

113 Wilder AP, Palumbi SR, Conover DO, Therkildsen NO. Footprints of local adaptation span hundreds of linked genes in the Atlantic silverside genome. Evol Lett. 2020;4(5):430-43. Epub 2020/10/06. doi: 10.1002/evl3.189. PubMed PMID: 33014419; PubMed Central PMCID: PMCPMC7523562.

114 Maroso F, Gkagkavouzis K, De Innocentiis S, Hillen J, do Prado F, Karaiskou N, et al. Genome-wide analysis clarifies the population genetic structure of wild gilthead sea bream (Sparus aurata). PLoS One. 2021;16(1):e0236230. Epub 2021/01/12. doi: 10.1371/journal.pone.0236230. PubMed PMID: 33428622; PubMed Central PMCID: PMCPMC7799848 competing financial interests or personal relationships that could have influenced the work reported in this paper. The commercial affiliation with Fios Genomics Ltd. does not alter our adherence to PLOS ONE policies on sharing data and materials.

115 Hollenbeck CM, Portnoy DS, Gold JR. Evolution of population structure in an estuarine-dependent marine fish. Ecol Evol. 2019;9(6):3141-52. Epub 2019/04/10. doi: 10.1002/ece3.4936. PubMed PMID: 30962887; PubMed Central PMCID: PMCPMC6434539.

116 Andrews KR, Good JM, Miller MR, Luikart G, Hohenlohe PA. Harnessing the power of RADseq for ecological and evolutionary genomics. Nat Rev Genet. 2016;17(2):81-92. Epub 2016/01/06. doi: 10.1038/nrg.2015.28. PubMed PMID: 26729255; PubMed Central PMCID: PMCPMC4823021.

117 Peterman W, Brocato, ER, Semlitsch RD, and Eggert LS. Reducing bias in population and landscape genetic inferences: the effects of sampling related individuals and multiple life stages. PeerJ. 2016;4(e1813) doi: 10.7717/peerj.1813.

118 Wang J. Effects of sampling close relatives on some elementary population genetics analyses. Mol Ecol Resour. 2018;18(1):41-54. doi: 10.1111/1755-0998.12708.

119 Toyama KS, Crochet PA, Leblois R. Sampling schemes and drift can bias admixture proportions inferred by structure. Mol Ecol Resour. 202;20(6):1769-1785. doi: 10.1111/1755-0998.13234.

120 Lowry DB, Hoban S, Kelley JL, Lotterhos KE, Reed LK, Antolin MF, et al. Breaking RAD: an evaluation of the utility of restriction site-associated DNA sequencing for genome scans of adaptation. Mol Ecol Resour. 2017;17(2):142-52. Epub 2016/11/20. doi: 10.1111/1755-0998.12635. PubMed PMID: 27860289; PubMed Central PMCID: PMCPMC5446919.

121 Powell A, Pooley CL, Scolamacchia M, Garzia de Leaniz C. Review of lumpfish biology. In: Treasurer JW, editor. Cleaner fish biology and aquaculture applications: 5M Publishing Ltd; 2018. p. 98-121.

122 Hedeholm RB, Post S, Grønkjær P. Life history trait variation of Greenland lumpfish (Cyclopterus lumpus) along a 1600 km latitudinal gradient. Polar Biology. 2017;40(12):2489-98. doi: 10.1007/s00300-017-2160-x.

123 COSEWIC. COSEWIC assessment and status report on the Lumpfish Cyclopterus lumpus in Canada. Committee on the Status of Endangered Wildlife in Canada. 2017.

124 Eriksen E, Durif CMF, Prozorkevich D. Lumpfish (Cyclopterus lumpus) in the Barents Sea: development of biomass and abundance indices, and spatial distribution. ICES Journal of Marine Science. 2014;71(9):2398-402. doi: 10.1093/icesjms/fsu059.

125 Durif CMF. Regulering av fisket etter rognkjeks. Havforskningsinstituttet, 2020.

126 GINR. Assessment of lumpfish (Cyclopterus lumpus) in West Greenland based on commercial data 2010-2021. Greenland Institute of Natural Resources, Nuuk. Technical report. pp 16. https://natur.gl/wp-content/uploads/2021/09/Assessment-of-lumpfish-Cyclopterus-lumpus-in-West-Greenland-2021.pdf 2021.

127 Ovenden JR, Berry O, Welch DJ, Buckworth RC, Dichmont CM. Ocean's eleven: a critical evaluation of the role of population, evolutionary and molecular genetics in the management of wild fisheries. Fish and Fisheries. 2015;16(1):125-59. doi: 10.1111/faf.12052.

128 VKM, Rueness E, Berg PR, Gulla S, Halvorsen K, Järnegren J, et al. Assessment of the risk to Norwegian biodiversity from import of wrasses and other cleaner fish for use in aquaculture. Opinion of the Panel on Alien Organisms and Trade in Endangered Species (CITES) of the Norwegian Scientific Committee for Food and Environment. . Oslo, Norway: Norwegian Scientific Committee for Food and Environment (VKM) 2019 Contract No.: 15.

129 Halvorsen K, Skiftesvik A, Durif C, Faust E, Wennhage H, André C, et al. Towards a sustainable fishery and use of cleaner fish in salmonid aquaculture: Challenges and opportunities. Report to Nordic Council of Ministers. . 2021.

130 Adlard RD, Miller TL, Smit NJ. The butterfly effect: parasite diversity, environment, and emerging disease in aquatic wildlife. Trends Parasitol. 2015;31(4):160-6. Epub 2014/12/10. doi: 10.1016/j.pt.2014.11.001. PubMed PMID: 25488771.

131 Blakeslee AMH, Manousaki T, Vasileiadou K, Tepolt CK. An evolutionary perspective on marine invasions. Evol Appl. 2020;13(3):479-85. Epub 2020/05/21. doi: 10.1111/eva.12906. PubMed PMID: 32431730; PubMed Central PMCID: PMCPMC7045714.

132 Faust, E. (2022). Crossing barriers: Genetic consequences of translocating wild cleaner fish for aquaculture. PhD Thesis. Gothenburg: University of Gothenburg. ISBN 978-91-8009-693-5. Electronic version available at http://hdl.handle.net/2077/70594

133 Jansson, E., Faust, E., Bekkevold, D., Quintela, M., Durif, C., Halvorsen, K.T., Dahle, G., Pampoulie, C.S., Kennedy, J., Whittaker, B., Unneland, L., André, C. & Glover, K.A. (2022). Global, regional, and cryptic genetic population structure in a high gene flow transatlantic fish. bioRxiv preprint. doi: 10.1101/2022.03.22.485384

Figure1

Click here to access/download;Figure;Fig1.tiff

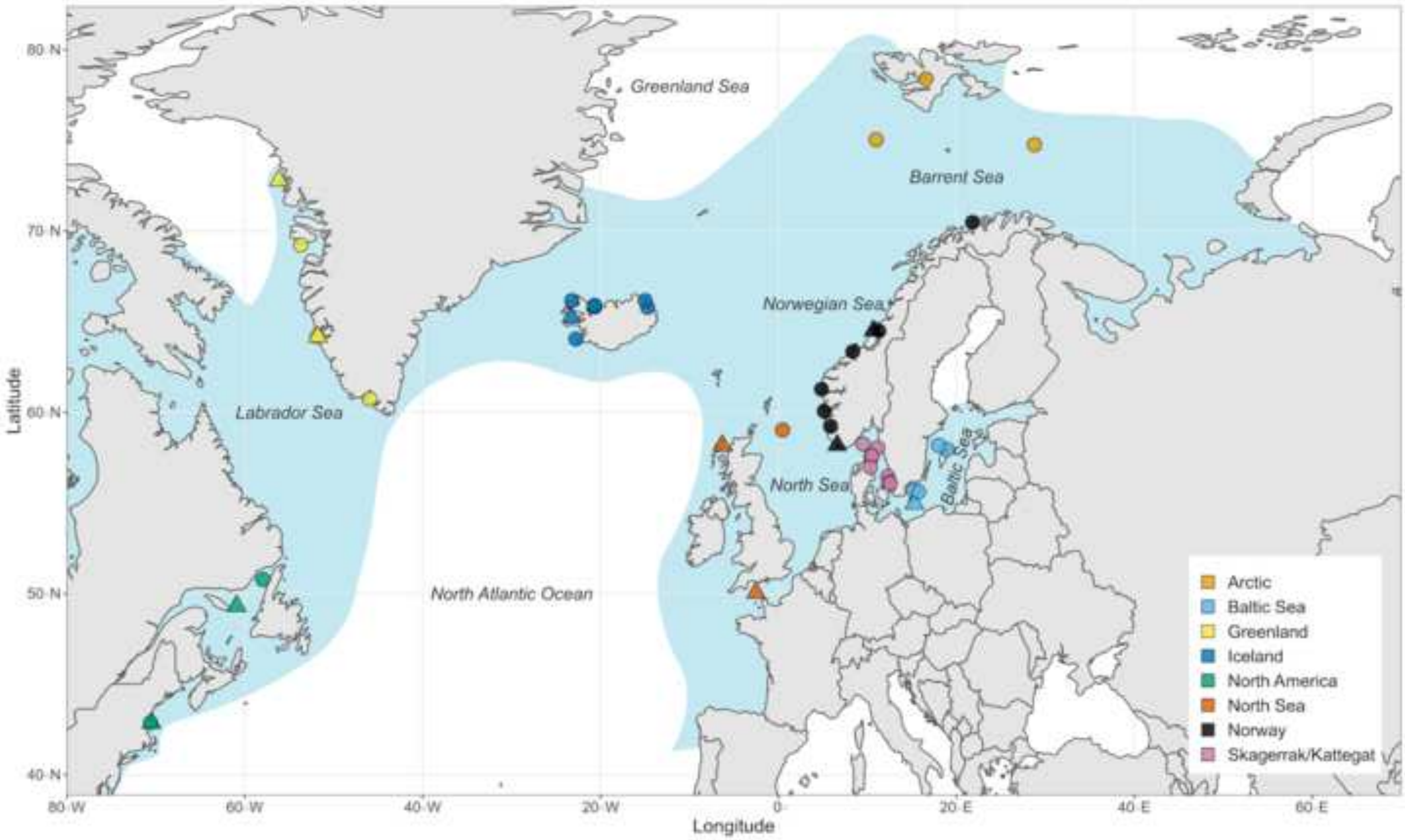

Figure2

Click here to access/download;Figure;Fig2.tiff

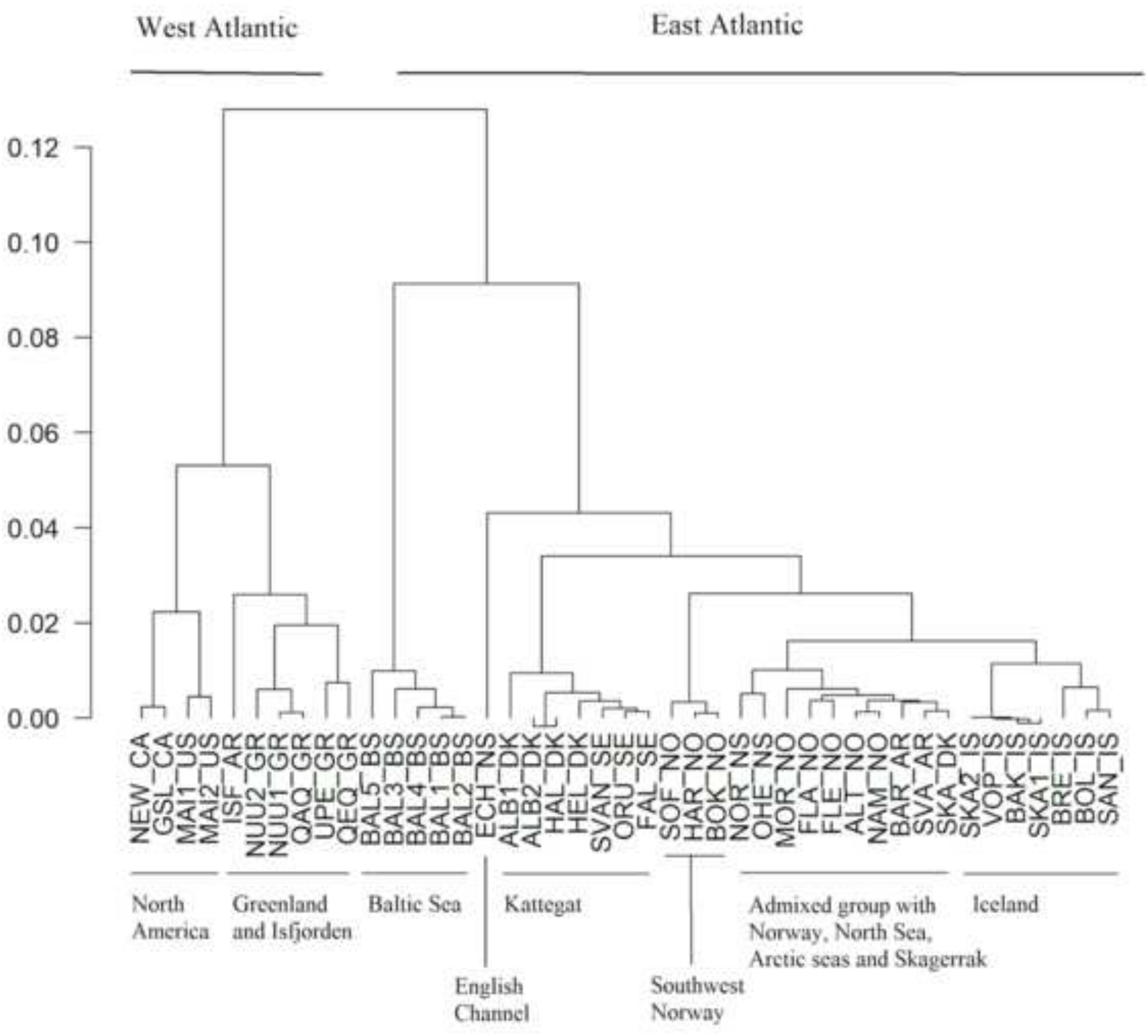

Figure3

Click here to access/download;Figure;Fig3.tiff

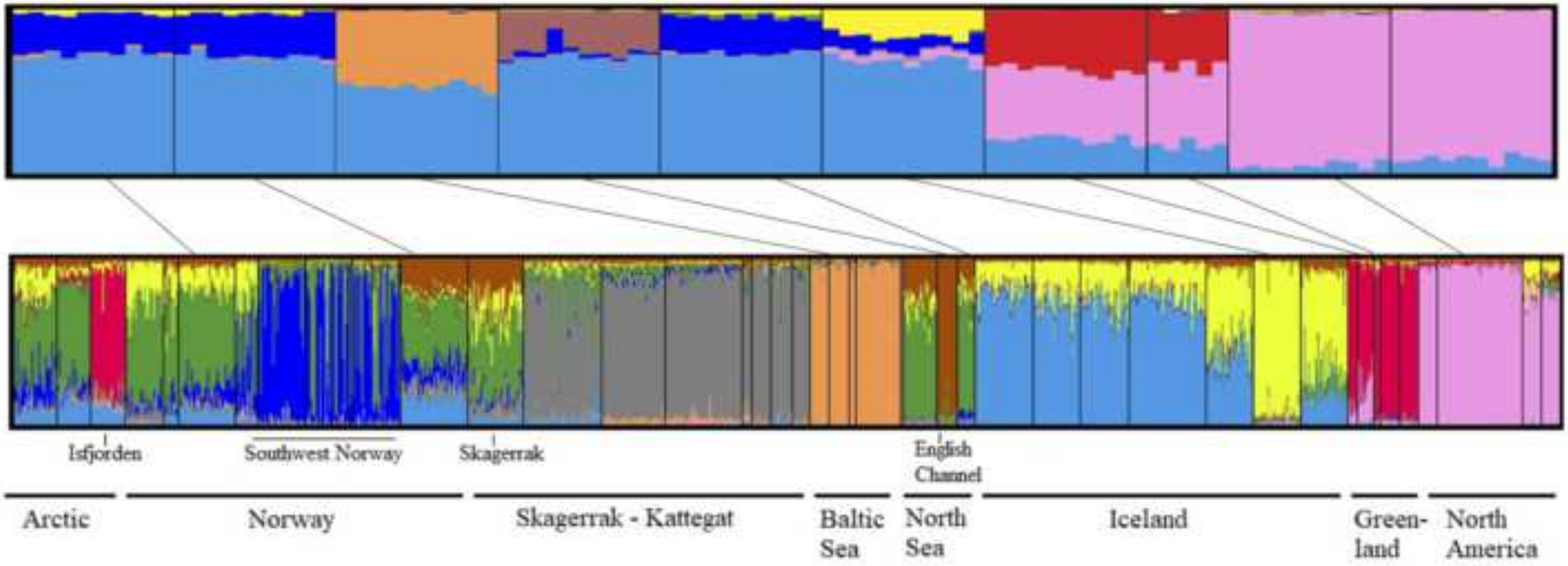

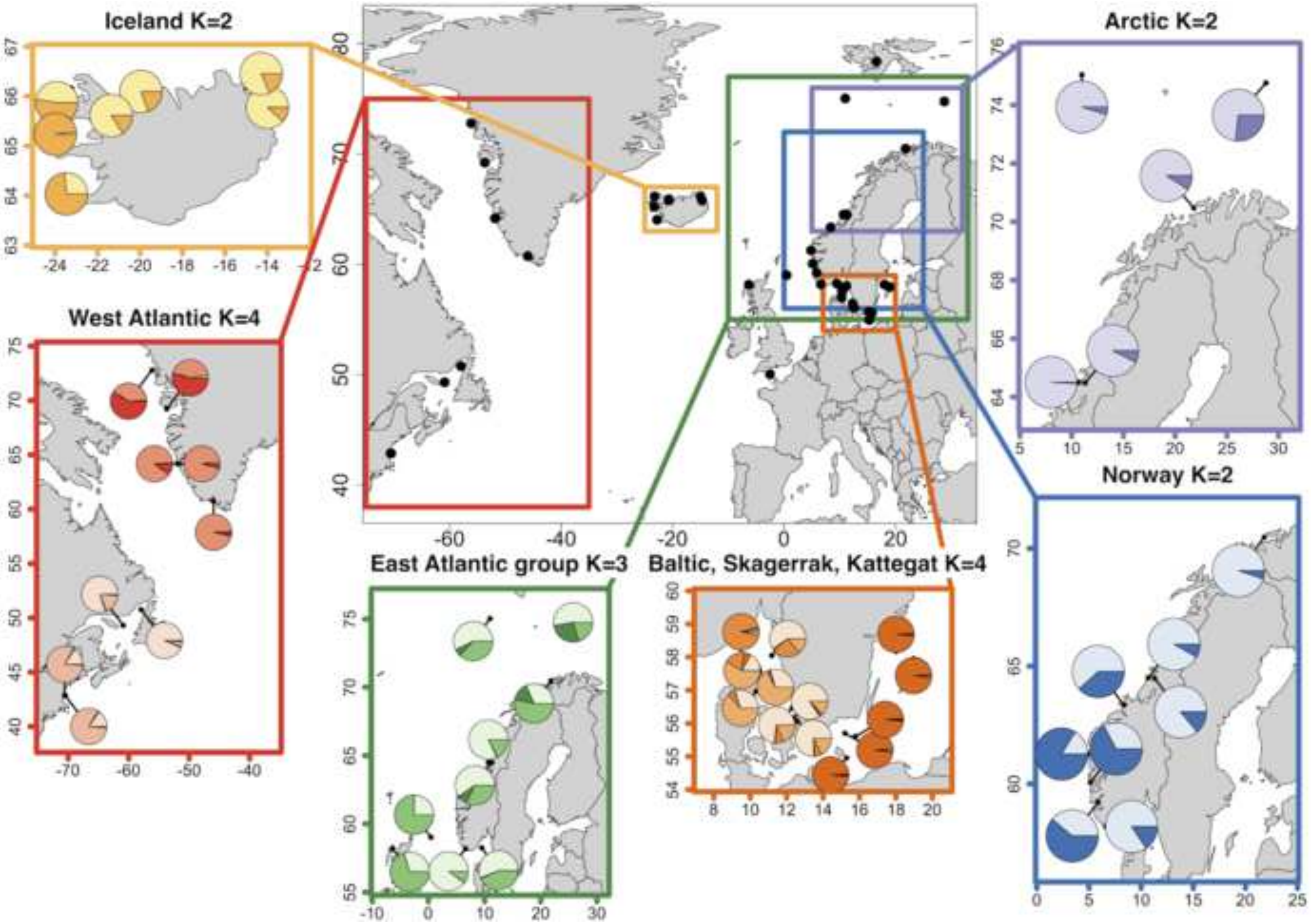
Iceland K=2
West Atlantic K=4
East Atlantic group K=3
Baltic, Skagerrak, Kattegat K=4
Arctic K=2
Norway K=2

Figure5

Click here to access/download;Figure;Fig5.tiff

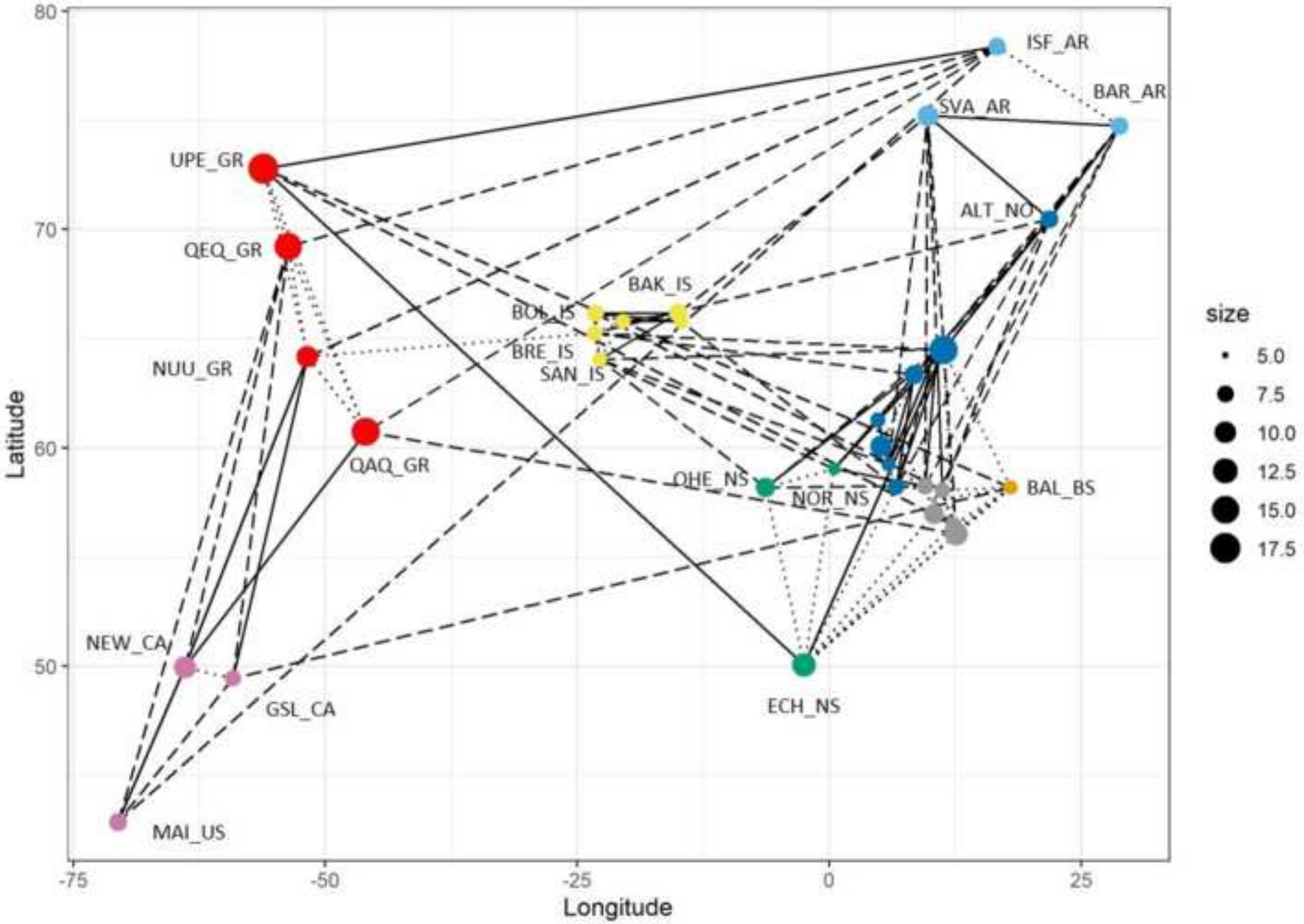

Figure6

Click here to access/download;Figure;Fig6.tiff

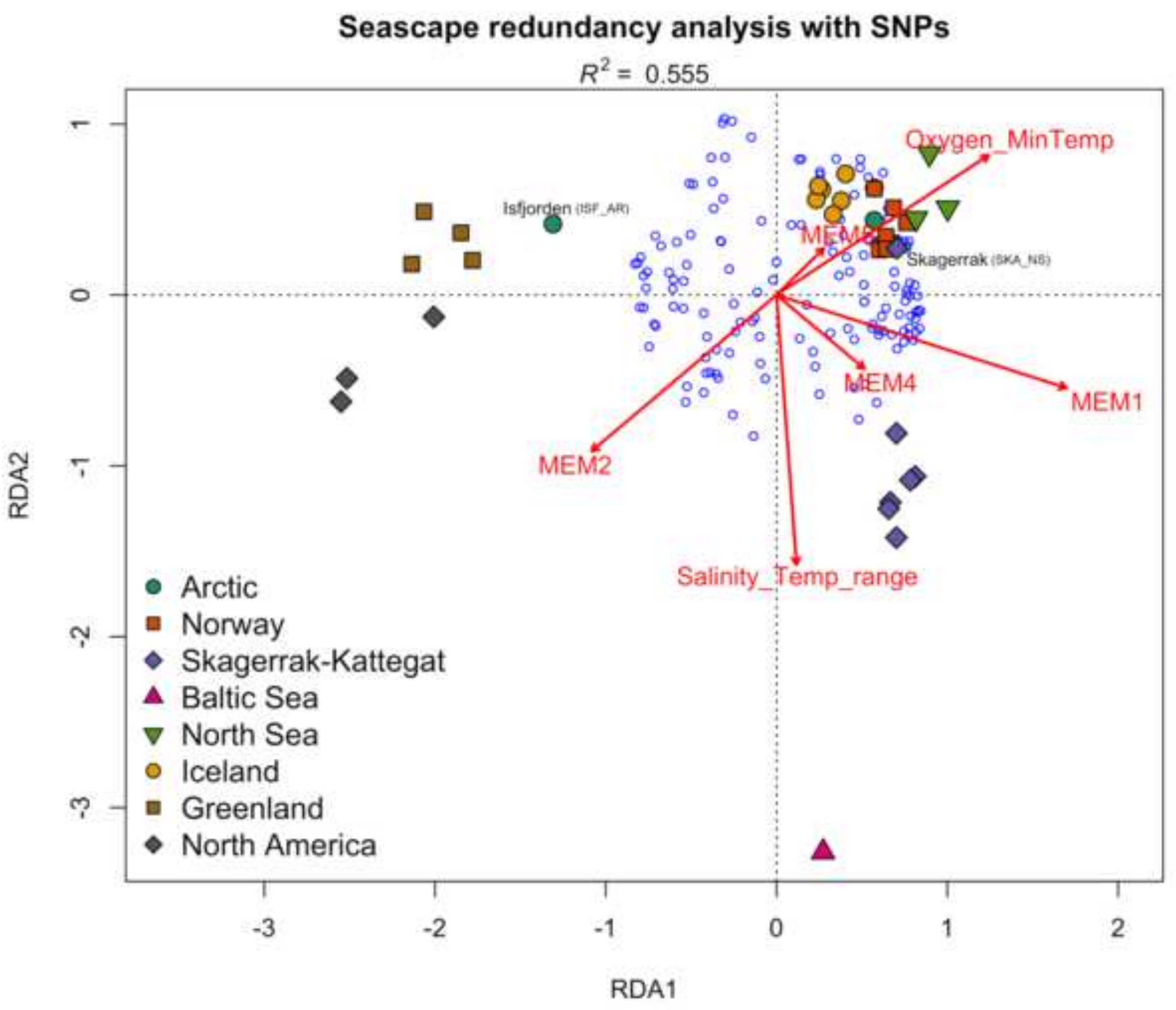

Click here to access/download
Supporting Information - Compressed/ZIP File Archive
Supplements.zip

Raw data

Click here to download Data Review URL
https://dataview.ncbi.nlm.nih.gov/object/PRJNA858951?reviewer=emj8d

Preprint PDF

Click here to access/download
Preprint PDF
Lumpfish_preprint.pdf
